# A Fungal Versatile GH10 Endoxylanase and Its Glycosynthase Variant: Synthesis of Xylooligosaccharides and Glycosides of Bioactive Phenolic Compounds

**DOI:** 10.3390/ijms23031383

**Published:** 2022-01-26

**Authors:** Ana Pozo-Rodríguez, Juan A. Méndez-Líter, Laura I. de Eugenio, Manuel Nieto-Domínguez, Eva Calviño, Francisco Javier Cañada, Andrés G. Santana, Jaime Díez, Juan L. Asensio, Jorge Barriuso, Alicia Prieto, María Jesús Martínez

**Affiliations:** 1 Department of Microbial & Plan Biotechnology, Centro de Investigaciones Biológicas Margarita Salas, Spanish National Research Council (CSIC), C/Ramiro de Maeztu 9, 28040 Madrid, Spain; ana.pozo@cib.csic.es (A.P.-R.); jmendez@cib.csic.es (J.A.M.-L.); lidem@cib.csic.es (L.I.d.E.); mnietdom@gmail.com (M.N.-D.); jaimediezrod94@gmail.com (J.D.); jbarriuso@cib.csic.es (J.B.); aliprieto@cib.csic.es (A.P.); 2 Department of Structural and Chemical Biology, Centro de Investigaciones Biológicas Margarita Salas, Spanish National Research Council (CSIC), C/Ramiro de Maeztu 9, 28040 Madrid, Spain; eva.calvino@cib.csic.es (E.C.); jcanada@cib.csic.es (F.J.C.); 3 CIBER de Enfermedades Respiratorias (CIBERES), Avda. Monforte de Lemos 3-5, 28029 Madrid, Spain; 4 Department of Bioorganic Chemistry, Instituto de Química Orgánica General, Spanish National Research Council (CSIC), C/Juan de la Cierva 3, 28006 Madrid, Spain; andres.g.santana@csic.es (A.G.S.); juanluis.asensio@csic.es (J.L.A.)

**Keywords:** antioxidants, fungal enzyme, glycosylation, hydrolysis, oligosaccharides, protein engineering

## Abstract

The study of endoxylanases as catalysts to valorize hemicellulosic residues and to obtain glycosides with improved properties is a topic of great industrial interest. In this work, a GH10 β-1,4-endoxylanase (XynSOS), from the ascomycetous fungus *Talaromyces amestolkiae*, has been heterologously produced in *Pichia pastoris*, purified, and characterized. rXynSOS is a highly glycosylated monomeric enzyme of 53 kDa that contains a functional CBM1 domain and shows its optimal activity on azurine cross-linked (AZCL)–beechwood xylan at 70 °C and pH 5. Substrate specificity and kinetic studies confirmed its versatility and high affinity for beechwood xylan and wheat arabinoxylan. Moreover, rXynSOS was capable of transglycosylating phenolic compounds, although with low efficiencies. For expanding its synthetic capacity, a glycosynthase variant of rXynSOS was developed by directed mutagenesis, replacing its nucleophile catalytic residue E236 by a glycine (rXynSOS-E236G). This novel glycosynthase was able to synthesize β-1,4-xylooligosaccharides (XOS) of different lengths (four, six, eight, and ten xylose units), which are known to be emerging prebiotics. rXynSOS-E236G was also much more active than the native enzyme in the glycosylation of a broad range of phenolic compounds with antioxidant properties. The interesting capabilities of rXynSOS and its glycosynthase variant make them promising tools for biotechnological applications.

## 1. Introduction

The use of lignocellulosic residues as a renewable source to synthesize high-value products plays a crucial role in the establishment of a circular economy [1]. Hemicellulose is one of the main components of these wastes, together with cellulose and lignin. The most abundant type of hemicellulose in plant biomass is xylan, which is present in dicotyledons and cereal grains [2]. This heteropolysaccharide consists of a β-(1–4)-linked d-xylopyranose backbone highly acetylated and branched by different side chains, which mainly include residues of arabinose, glucuronic acid, and ferulic acid [3,4]. Endoxylanases (EC 3.2.1.8) are considered the most important biocatalysts of xylan hydrolysis, since they start its breakdown by randomly hydrolyzing the β-1,4 linkages between the xylopyranose units of the backbone, releasing xylooligosaccharides (XOS) that are further hydrolyzed to xylose by β-xylosidases [4]. However, depending on xylan’s complexity, other enzymes such as α-arabinofuranosidases, α-glucuronidases, feruloyl esterases, and acetylxylan esterases are necessary to complete its degradation [4,5].

The CAZy database classifies the enzymes active on carbohydrates, describing the families of structurally related proteins that degrade, modify, or create glycosidic bonds [6]. Endoxylanases are glycosyl hydrolases (GH) widely distributed among CAZy families, of which families GH10 and GH11 are the most studied. Endoxylanases from these two groups share the retaining mechanism of hydrolysis; however, they differ in their structural, physicochemical, and catalytic properties [7]. GH10 enzymes display a (β/α)_8_ barrel fold and have higher molecular mass (>30 kDa) and lower isoelectric point than their GH11 counterparts, which are characterized by showing a β-jelly roll fold and a wide range of pH tolerance [7,8,9]. Furthermore, GH10 enzymes exhibit a broad substrate specificity, which makes them more effective in the hydrolysis of heteroxylans. This versatility is explained by their capacity to hydrolyze β-1,4 linkages between xylopyranose residues located in the surroundings of side chains and by their ability to break down β-1,3 glycosidic bonds in the main backbone and even low molecular mass cellulosic substrates [4,8,10]. In contrast, GH11 endoxylanases are very specific, hydrolyzing exclusively β-1,4 linkages and being inhibited by the proximity of substituents and β-1,3 glycosidic bonds [8].

There is great industrial interest in the study of endoxylanases given their broad range of applications, and their potential to hydrolyze hemicellulose and for plant waste utilization. First, endoxylanases can be employed in the saccharification of lignocellulosic residues both to obtain xylose from xylan and as processing aids, since the degradation of hemicellulose facilitates the accessibility of other hydrolytic enzymes to cellulose [11]. In addition, endoxylanases are able to convert xylan into high-value products such as XOS of different lengths (2 to 10 xylopyranose units), which are known to be emerging prebiotics [7,12]. Moreover, these enzymes have been extensively used in the pulp and paper industry to improve bleaching because they contribute to breaking down the xylan network, which helps to remove trapped lignin, reducing the consumption of bleaching chemicals and the environmental impact of the process [13,14]. Finally, endoxylanases enhance feed digestibility and food properties when included as additives [15].

Although the main activity of GHs is based on their hydrolytic capacity, it has been demonstrated that some of these enzymes display the capacity of transferring glycosyl residues to acceptor compounds [16]. This reaction is called transglycosylation and represents a powerful biotechnological tool to modify molecules of interest with the aim of improving their properties. For instance, the glycosylation of phenolic antioxidants can increase their solubility, stability, bioavailability, and safety [17]. In addition, the synthesis of oligosaccharides of different degrees of polymerization has also been achieved by this mechanism [18]. The enzymatic synthesis of glycosides has arisen as an environmentally friendly and efficient alternative to chemical synthesis as it enables an easier control of stereo- and regioselectivity, avoids the need for protecting groups strategies, and facilitates the use of mild reaction conditions [19,20]. Nevertheless, transglycosylation yields with GHs are usually low because the glycosylated products are substrate for hydrolysis. To make these reactions economically feasible, the hydrolytic capacity of GHs should be eliminated or reduced [16]. Directed mutagenesis approaches lead to the creation of two major enzyme variants, glycosynthases and thioglycoligases, by replacing one of the catalytic residues (nucleophile or acid/base, respectively) in retaining GHs by a non-participating amino acid [21].

Endoxylanases are produced by a wide variety of microorganisms (fungi, bacteria, yeast, algae, protozoans, snails, crustaceans, insect, seeds, etc.), of which filamentous fungi are the preferred source thanks to their higher secretion and activity levels [5,22,23]. In this regard, *Talaromyces amestolkiae* [24], an ascomycete isolated from cereal residues, was selected after a previous fungal screening for its ability to produce high levels of cellulases, xylanases, and other GHs [12,25,26]. Genomic analyses revealed several genes coding for putative endoxylanases, being the GH11 endoxylanase XynM the only one characterized so far in this ascomycete [12]. This endoxylanase exhibited the capacity to break down xylan into a mixture of XOS with prebiotic effects that stimulated the growth of beneficial bacteria in the lower gastrointestinal tract and prevented the development of potential pathogenic species. In addition, the recombinant XynM, produced in *Pichia pastoris* (synonym *Komagataella pastoris* [27], one of the most utilized hosts for heterologous protein production [28]), was used in an enzymatic cascade with the β-xylosidase rBxTW1 of the same fungus to synthesize an antiproliferative xyloside from xylan [29].

In this work, a new β-1,4-endoxylanase (XynSOS) from the GH10 family, identified in the genome of *T. amestolkiae*, was successfully expressed in *P. pastoris.* The purification and in-depth characterization of the recombinant enzyme revealed its versatility to hydrolyze a wide range of substrates and a high affinity for beechwood xylan and wheat arabinoxylan. In addition, a glycosynthase variant was developed by directed mutagenesis, showing enhanced glycosylation yields and the potential to synthesize XOS of different lengths.

## 2. Results and Discussion

### 2.1. Cloning, Production, and Purification of rXynSOS

A putative enzyme from the GH10 family, called g9427 and re-named as β-1,4-endoxylanase XynSOS, was detected in very low amounts when *T. amestolkiae* was grown in Mandels medium with microcrystalline cellulose, slurry, and xylan as carbon sources [25]. The *xynSOS* gene was located in the genome of the fungus [25] comprising 1445 bp, 3 introns, and a signal peptide region coding for 19 amino acids (Figure S1). The mature XynSOS protein is composed of 389 amino acids and contains a catalytic domain that shows high identity with other GH10 enzymes (HMMER dbCAN2, *E*-value 3.6 × 10^−98^) and a *C*-terminal Carbohydrate-Binding Module (CBM) assigned to family 1 (HMMER dbCAN2, *E*-value 8.4 × 10^−17^) and connected by a Ser/Thr rich linker region (Figure S2), as previously described in other GH10 endoxylanases [30].

**Figure 1:**
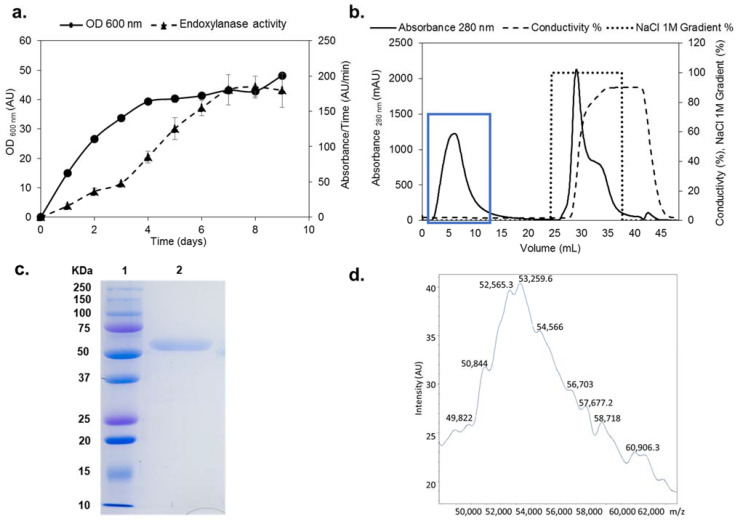
Production, purification, and characterization of rXynSOS. (**a**) Endoxylanase activity against AZCL–beechwood xylan and OD_600 nm_ of the selected rXynSOS *P. pastoris* clone grown for 9 days in YEPS medium with methanol induction. (**b**) Purification of rXynSOS from *P. pastoris* crude extracts by anion-exchange chromatography. Protein absorbance through the NaCl gradient was monitored. The enzyme was purified in one single step. The blue rectangle indicates the peak of enzyme elution. (**c**) Determination of rXynSOS estimated molecular mass (Mw) by SDS-PAGE with the molecular weight marker displayed in lane 1. (**d**) MALDI-TOF spectrum of rXynSOS showing the profile of a glycosylated protein.

The *xynSOS* gene without the three introns and lacking the signal peptide was expressed in *P. pastoris* GS115, since several GHs from this fungus have been successfully expressed in this model yeast to increase their production and facilitate their purification [26]. The clone with the highest recombinant XynSOS (rXynSOS) expression was selected for production, reaching a maximal endoxylanase activity against AZCL–beechwood xylan of 180 AU/min in the 9th day of culture in YEPS liquid medium (Figure 1a). The protein was purified from the crude extracts of *P. pastoris* in one single step using anion-exchange chromatography (Figure 1b), obtaining high-purification yields (recovered activity: 72%).

### 2.2. Physicochemical Properties of rXynSOS

The molecular mass (*M*_w_) of rXynSOS determined by SDS-PAGE (Figure 1c) was around 50 kDa. This *M*_w_ is higher than the calculated by the ProtParam server from the rXynSOS amino acid sequence (41.57 kDa), which is probably due to the higher glycosylation levels of *P. pastoris* proteins [31]. The accurate *M*_w_ of the enzyme determined by MALDI-TOF was around 53 kDa (Figure 1d), showing a wide signal with multiple poorly defined peaks. This heterogeneous pattern is due to glycosylation, since 32 sites of *O*-glycosylation and one site of *N*-glycosylation were predicted in rXynSOS from its amino acid sequence (Figure S2). The *M*_w_ calculated by size exclusion chromatography was 55 kDa, indicating that rXynSOS is a monomeric protein in solution.

The maximum endoxylanase activity against AZCL-beechwood as substrate was observed at pH 5 and 70 °C (Figure S3). These values are comparable to those described for other fungal GH10 endoxylanases, ranging between 60 and 80 °C for the optimal temperature and pH 4.5 and 6 for the optimal pH [32,33,34,35,36,37].

The analysis of the functionality of the predicted rXynSOS CBM1 domain carried out after up to 2 h incubation of the enzyme with microcrystalline cellulose showed that the endoxylanase activity in the supernatant decreased very quickly, remaining only 27% after 10 min and 15% after 1 h, respectively. This result indicates that this CBM1 domain is functional and allows the enzyme to strongly bind to cellulose. Previous studies have postulated that the CBM1 domain enables GH10 endoxylanases to approach lignocellulosic biomass and bind to its crystalline cellulose, facilitating the degradation of the surrounding xylan [30,38].

### 2.3. Substrate Specificity and Kinetics of rXynSOS

The potential of rXynSOS to break down a broad range of substrates is displayed in Table 1. The enzyme was highly active against beechwood xylan (132.33 U mg^−1^) and especially against wheat arabinoxylan (149.15 U mg^−1^). This small preference for branched over linear xylans has also been reported for the GH10 endoxylanase XynD from *Penicillium funiculosum* [34]. rXynSOS was also able to hydrolyze CMC, although much less efficiently than the xylan substrates, as it was described with the GH10 endoxylanase AFUMN-GH10 from *Aspergillus fumigatus* [32]. Nevertheless, the enzyme could not hydrolyze microcrystalline cellulose and cellobiose, which are other specific substrates for enzymes with cellulolytic activity. The analysis on *p*-nitrophenyl-derived sugars revealed that rXynSOS can break down all four that were tested, being highly active on *p*NPX_2_ but showing lower hydrolytic activity against *p*NPX, *p*NPG_2_, and *p*NPG. As expected, a tendency to hydrolyze longer substrates was observed, since endoxylanase activity was better with xylans, and it was also higher on *p*NPX_2_ and *p*NPG_2_ than on *p*NPX and *p*NPG, respectively.

Overall, these results confirm rXynSOS substrate versatility, which is in agreement with the capabilities of GH10 endoxylanases to hydrolyze β-1,4 linkages between xylopyranose residues located in the surroundings of side chains and even some cellulosic substrates [4,8,10]. In contrast, GH11 endoxylanases are usually very specific, as is the case of the endoxylanase XynM from the same *T. amestolkiae* strain, which was able to degrade beechwood xylan but no other substrates such as *p*NPX, *p*NPG, and CMC [12].

The kinetic parameters calculated for the best rXynSOS substrates, as well as for CMC as a representative of cellulose activity, are displayed in Table 2. The enzyme showed a high affinity for beechwood xylan and wheat arabinoxylan, with *K_m_* values of 1.07 and 1.94 g L^−1^, respectively, which are better than the ones reported for most fungal GH10 endoxylanases [32,34,36,37]. The catalytic efficiencies (*k_cat_*/*K_m_*) of rXynSOS on beechwood xylan (134.69 s^−1^ g^−1^ L) and wheat arabinoxylan (84.65 s^−1^ g^−1^ L) are in the same range as those of the GH10 endoxylanases Xyn10A from *Penicillium oxalicum* [36], PspXyn10 from *Penicillum* sp. [37], and XynD from *P. funiculosum* [34]. However, GH10 endoxylanases Xyn10B from *P. oxalicum* [36] and AFUMN-GH10 from *A. fumigatus* [32] exhibit unusually higher activities on beechwood xylan. In addition, the highest affinity (0.12 mM, 0.05 g L^−1^) and catalytic efficiency (880.04 s^−1^ mM^−1^, 2181.45 s^−1^ g^−1^ L) of rXynSOS were obtained with *p*NPX_2_ as substrate. Regarding the kinetic difference of GH10 and GH11 endoxylanases from *T. amestolkiae*, the results indicate that rXynSOS (GH10) is much more efficient than XynM (GH11) in the hydrolysis of beechwood xylan [12], in which the *k_cat_*/*K_m_* values are 134.69 and 7.76 s^−1^ g^−1^ L, respectively.

### 2.4. Transglycosylation Potential of rXynSOS

The transglycosylation capacity of rXynSOS was assayed using *p*NPX_2_ as a xylobiose donor and vanillyl alcohol, 2-hydroxybenzyl alcohol, hydroquinone, and gallic acid as acceptors chosen on the basis of their interesting antioxidant properties and other health benefits [39,40] (Figure S4). The results showed that rXynSOS was able to transglycosylate all the acceptors assayed, but with low efficiencies, since the formation of glycosides was barely detectable by TLC and only confirmed by ESI-MS (Figure S5). Considering the wide substrate specificity observed in the enzyme’s hydrolytic activity, *p*NPX, *p*NPG, and *p*NPG_2_ were tested as donors of xylose, glucose, and cellobiose, respectively, for the transglycosylation of vanillyl alcohol. Despite the low efficiencies, ESI-MS spectra disclosed the production of glycosides for all the selected donors (Figure S6).

Although rXynSOS can transglycosylate different phenolic compounds employing a broad spectrum of sugar donors, its efficiency needs to be increased to enable its biotechnological application. The optimization of reaction conditions toward an enhanced transglycosylation/hydrolysis ratio is one of the strategies to improve glycoside yields [41]; however, the glycosynthase strategy based on eliminating the hydrolytic activity of the enzymes through protein engineering has been proven to be a better approach [20,41]. Therefore, a novel glycosynthase derived from rXynSOS was designed.

### 2.5. Conversion of rXynSOS into Its Glycosynthase Variants

Two glycosynthase variants of rXynSOS were developed by replacing its nucleophile catalytic residue by an inert one. The identification of rXynSOS nucleophile residue, a glutamic acid at position 236, was carried out via Clustal Omega alignment of XynSOS amino acid sequence with the sequences of three well-characterized fungal GH10 endoxylanases (Figure S7). Then, this residue was substituted by directed mutagenesis by glycine (rXynSOS-E236G) and serine (rXynSOS-E236S), since these mutations have been reported to generally produce higher glycosylation yields [18,42].

**Figure 2:**
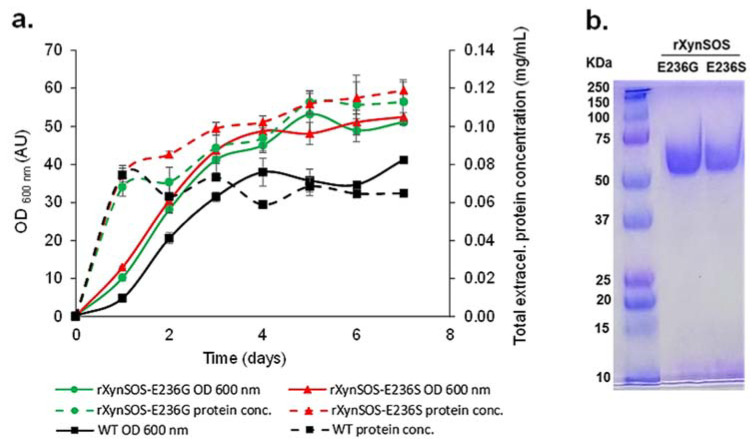
Production and purification of rXynSOS-E236G and rXynSOS-E236S glycosynthase variants. (**a**) Total extracellular protein concentration and OD_600 nm_ of wild-type (WT) *P. pastoris* and the selected rXynSOS-E236G and rXynSOS-E236S-producing clones. Cultures were grown for 7 days in YEPS medium with methanol induction. (**b**) SDS-PAGE analysis of purified rXynSOS-E236G (lane 2) and rXynSOS-E236S (lane 3) variants. Molecular weight marker is displayed in lane 1.

The two novel rXynSOS glycosynthase variants, which need activated glycosyl donors such as glycosyl fluorides to act on and catalyze the glycosylation reactions [19], were produced in *P. pastoris* GS115, selecting the clones by their higher total extracellular protein concentrations, since they no longer have hydrolytic activity (Figure 2a). The glycosynthase variants were purified in one single step using anion-exchange chromatography, following the same protocol as for the native rXynSOS, and their purity was confirmed by SDS-PAGE (Figure 2b).

**Figure 3:**
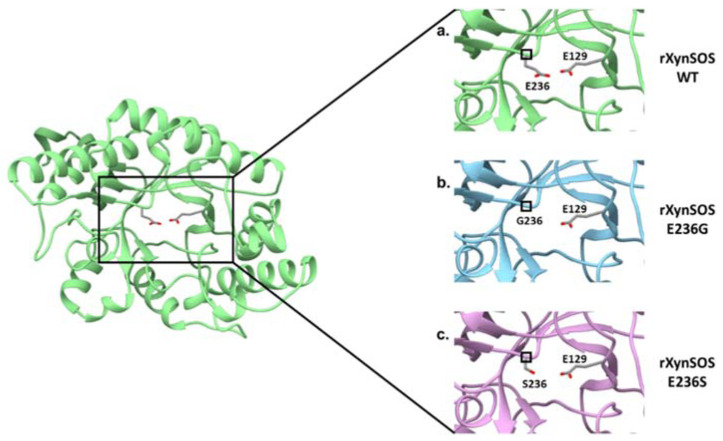
Conversion of *T. amestolkiae* rXynSOS into the glycosynthase variants rXynSOS-E236G and rXynSOSE236S. The three-dimensional model of the rXynSOS GH10 domain was obtained with SWISS-MODEL. The server selected as a template the PDB structure 6Q8M (GH10 endoxylanase from *Aspergillus aculeatus*), which has a sequence identity of 65.35% and resulted in a model of QMEAN 0.86. The catalytic amino acids E236 and E129 are displayed (**a**). Substitutions of the nucleophile catalytic residue E236 for G236 (lacking the side chain, **b**) and for S236 (shorter side chain, **c**) are shown to illustrate their effect in eliminating or reducing the hydrolytic capacity of the enzyme.

The glycosylation activity of rXynSOS glycosynthase variants was studied in reactions with X_2_F as donor and *p*NPX_2_ as acceptor. Most of the glycosynthases that have been described are able to use *p*NP sugars as good acceptors for glycosylation, giving rise to *p*NP-oligosaccharides of different lengths [18]. Surprisingly, *p*NPX_2_ was not a good acceptor molecule for neither of the glycosynthase variants. In the case of rXynSOS-E236S, no products were detected either by TLC or ESI-MS, while for rXynSOS-E236G, the synthesis of *p*NPX_4_ and *p*NPX_6_ could only be confirmed by ESI-MS (Figure S8a) but not by TLC. To generate more conclusive data, we performed the reactions with vanillyl alcohol as an alternative acceptor molecule. The TLC results showed the expected glycosylated product for rXynSOS-E236G (see below, Section 2.7), which was also confirmed by ESI-MS (Figure S8b). Nevertheless, rXynSOS-E236S was, again, unable to glycosylate the acceptor. The same behavior has been observed with other glycosynthases, being the synthetic activity of the serine mutants very low compared to the one of glycine variants [43]. This could be explained considering previous studies, which indicated that the rigid serine side chain might be an obstacle in the departure of the fluorine coming from the glycosyl fluorides. Moreover, the lack of side chain in glycine could also lead to a reduced steric hindrance, hosting the reaction more efficiently [18]. The detail of the catalytic nucleophile residue in the three-dimensional model of rXynSOS supports this hypothesis (Figure 3). Based on these results, the rXynSOS-E236G glycosynthase variant was selected for the following experiments.

### 2.6. Oligosaccharides Synthesis by the Glycosynthase Variant rXynSOS-E236G

**Figure 4:**
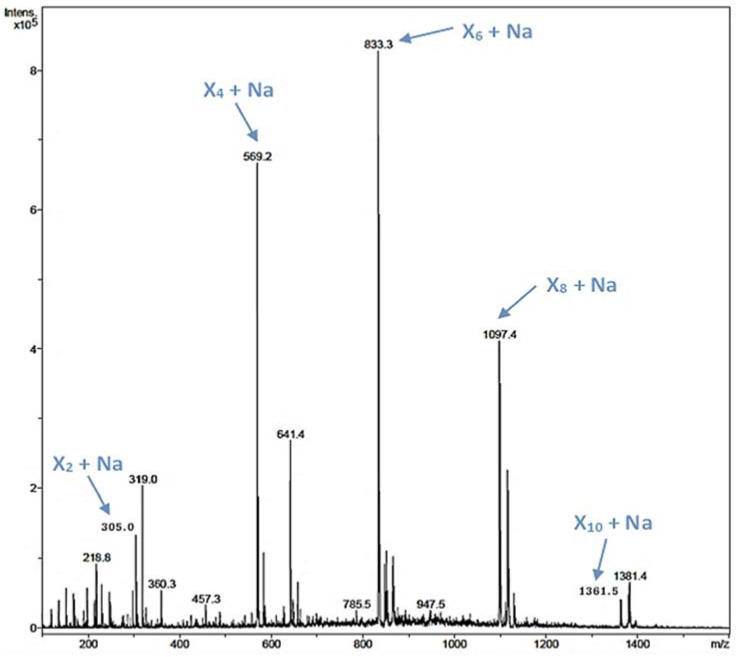
ESI-MS analysis of a reaction containing 20 mM X_2_F as the only reactant molecule and the glycosynthase variant rXynSOS-E236G as the catalyst. The *m*/*z* of ions corresponding to the Na adducts of xylooligosaccharides (XOS) of four, six, eight, and 10 xylose units (X_4_, X_6_, X_8_, and X_10_) are indicated by blue arrows.

Xylooligosaccharides (XOS) have been described as emerging prebiotics that have important health benefits, such as immunomodulatory, antitumoral, and antimicrobial activities [44]. XOS are obtained by chemical or enzymatic methods, being the enzymatic approach the preferred one [18,45]. In the rXynSOS-E236G reaction using *p*NPX_2_ as an acceptor and X_2_F as a donor described before, several peaks of mass corresponding to XOS were detected by ESI-MS (Figure S8a). Taking into account this finding, we hypothesized that the synthesis of XOS was favored over the formation of *p*NP-XOS. To further study this, we conducted a reaction with X_2_F as the only reactant molecule (no acceptor was added), in which the synthesis of XOS of different lengths (four, six, eight, and ten xylose units) was confirmed by ESI-MS (Figure 4). This XOS reaction was also monitored by Nuclear Magnetic Resonance (NMR) directly while it progressed in the NMR tube. The spectra of the reaction mixture in the different time points, when compared with the spectrum of a commercial 1,4-β-d-xylotetraose, corroborate the β-1,4 regioselectivity of rXynSOS-E236G in XOS synthesis (Figure S9). Unlike the XOS generated from xylan hydrolysis, which usually contain ramifications, the enzymatic oligomerization of X_2_F gives rise to linear chain XOS. This type of XOS can be of biotechnological interest, because they are not frequent in nature [19].

Previous reports illustrate XOS production by the enzymatic hydrolysis of xylan using, for instance, the GH11 endoxylanase XynM from *T. amestolkiae* [12] and the endoxylanases BLf1 from *Aspergillus brasiliensis*, rXynC from *P. funiculosum* [46], rPoXyn3 from *Penicillium occitanis* [47], and rT-XynC(122)C(166) from *Talaromyces thermophilus* [48]. However, the application of glycosynthases to produce XOS is a promising solution, since these enzymes do not hydrolyze the XOS obtained, which increases the efficiency of the reactions [18]. Although XOS synthesis from X_2_F has also been described for bacterial glycosynthases, such as those from the GH10 endoxylanases XylB from *Thermotoga maritima*, XynB from *Clostridium stercorarium*, XynA from *Bacillus halodurans*, and Cex from *Cellulomonas fimi* [42,43], rXynSOS-E236G is the first fungal glycosynthase reported with this capacity.

In order to check if the glycosynthase variant rXynSOS-E236G could synthesize a wider spectrum of oligosaccharides and also to verify that other sugar donors could be employed, several reactions using XF, GF, and G_2_F as xylose, glucose, and cellobiose donors, respectively, were carried out. ESI-MS results confirmed the formation of XOS using XF as a donor (Table S1), but of shorter lengths (two, three, and four xylose units) and less efficiently compared to the ones obtained with X_2_F. The reactions with the glucose-based donors were also less efficient, as no oligosaccharides were detected with GF, and only cellotetraose was observed when employing G_2_F. These findings are consistent with the hydrolytic efficiencies displayed in Table 1 for rXynSOS against *p*NPX, *p*NPG and *p*NPG_2_, in which *p*NPX_2_ was clearly preferred over them.

### 2.7. Glycosylation Profile of the Glycosynthase Variant rXynSOS-E236G

**Figure 5:**
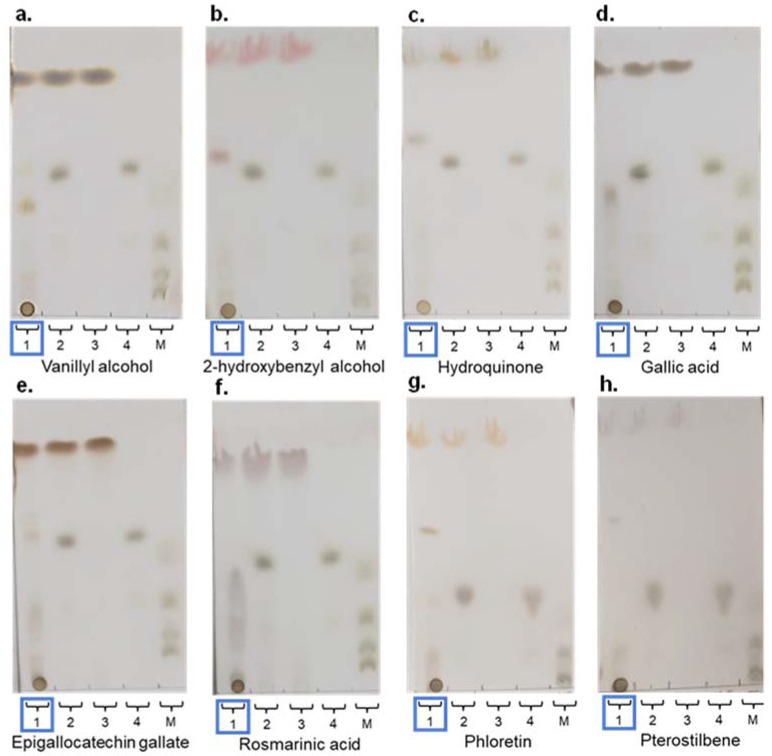
Thin layer chromatography (TLC) analysis of glycosylation reactions catalyzed by rXynSOS-E236G, using 20 mM X_2_F as donor and different acceptor molecules at 20 mM: (**a**) vanillyl alcohol; (**b**) 2-hydroxybenzyl alcohol; (**c**) hydroquinone; (**d**) gallic acid; (**e**) epigallocatechin gallate (EGCG); (**f**) rosmarinic acid; (**g**) phloretin; and (**h**) pterostilbene. Lane 1 (highlighted with a blue rectangle): samples of glycosylation mixtures containing the acceptor indicated in every case, X_2_F as xylobiose donor, the catalyst, and the reaction products. Lane 2: negative control with acceptor and X_2_F and no catalyst. Lane 3: negative control containing only the acceptor molecule. Lane 4: negative control containing only the donor X_2_F. Lane 5. Standards mixture with xylose, xylobiose, xylotriose, and xylotetraose.

The same molecules tested with the native rXynSOS were used as acceptors for the glycosynthase variant E236G (vanillyl alcohol, 2-hydroxybenzyl alcohol, hydroquinone, and gallic acid). This time, potential glycosides were clearly identified by TLC for all the phenolic compounds assayed (Figure 5a–d), and their molecular masses as sodium adducts were confirmed by ESI-MS. Table 3 shows the molecular mass of the xylobiosides detected in these reactions, although small peaks corresponding to xylotetraosides were also observed. In this regard, as all the acceptors tested have more than one hydroxyl group to potentially participate in the glycosylation reaction, it could be possible to have different xylobiosides as well as glycosides with one xylotetraose chain or two xylobiose chains located in different positions giving the same molecular mass. Overall, the results demonstrate that the glycosynthase variant rXynSOS-E236G is much more efficient than the native enzyme in glycosylation reactions.

In addition, more complex molecules such as the polyphenols epigallocatechin gallate (EGCG), rosmarinic acid, phloretin, and pterostilbene (Figure S4) were assayed as possible rXynSOS-E236G acceptors using X_2_F as the donor. These polyphenols are extracted from plants and exhibit numerous bioactive properties, including antioxidant, antihypertensive, antitumoral, bactericidal, neuroprotective, and anti-inflammatory activities [49]. Potential glycosides were detected by TLC for all these polyphenols (Figure 5e–h), and ESI-MS studies of their reaction products indicated the presence of different glycosides harboring xylobiose (Table 3) and others containing xylotetraose.

To further study these glycoside mixtures, HPLC analyses were carried out. One major peak as the main glycosylated product was observed for all the acceptors except for rosmarinic acid, for which two main xylobiosides were noticed (data not shown). Additionally, minor peaks were also detected, probably corresponding to glycosides containing the xylobiose in a different position or even a xylotetraose moiety. For phloretin, pterostilbene, and especially rosmarinic acid, more minor peaks and thereby wider glycoside mixtures were obtained as the result of using more complex acceptor molecules that include several potentially glycosylable hydroxyl groups.

The yield of the main glycoside in each reaction relative to the initial acceptor concentration was also calculated from the HPLC chromatograms, achieving the highest values with hydroquinone (26.0%), gallic acid (21.7%), and rosmarinic acid (two major peaks of 20.9% and 20.0%, which represents a 40.9% acceptor conversion) (Table 3). However, it must be noted that in the case of phloretin and pterostilbene, some precipitation of the acceptor molecules was observed during the reactions due to their low solubility under the conditions tested [50,51], which could have caused an underestimation of glycoside yields. These results confirm that rXynSOS-E236G glycosynthase is a promising tool to glycosylate a broad range of phenolic compounds with biotechnological applications. Furthermore, the high-glycoside yields obtained with some of the acceptors assayed could be further increased through the optimization of reaction conditions by, for instance, using multiparametric models that could determine the best values for the reaction parameters (i.e., acceptor, donor, and enzyme concentrations, time, etc.) [29,52,53].

**Figure 6:**
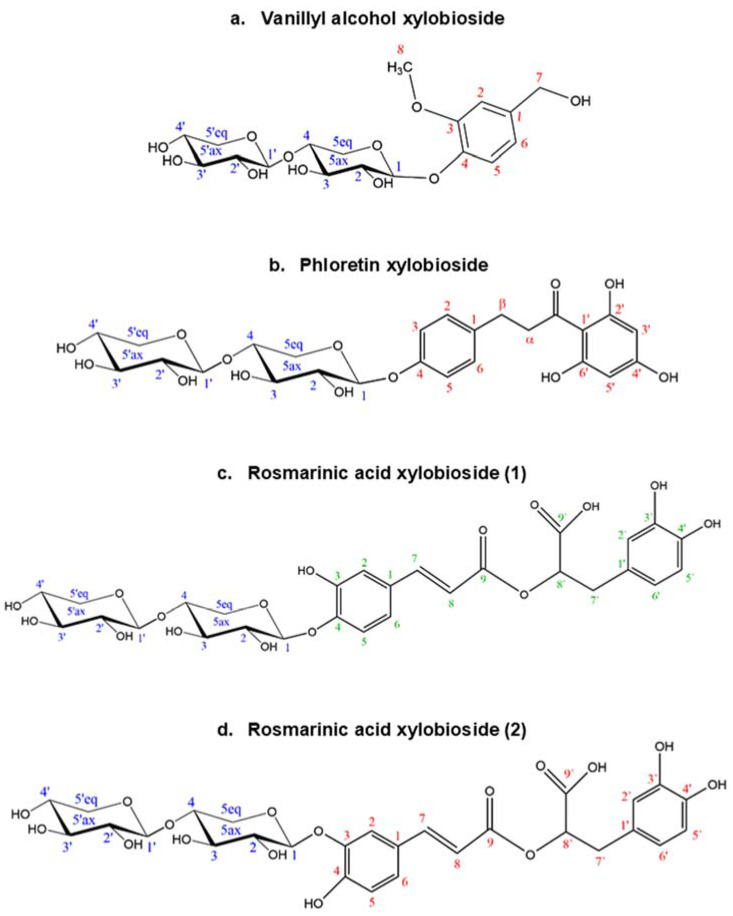
Structures deduced from NMR analysis of the glycosides of (**a**) vanillyl alcohol, (**b**) phloretin, and (**c**,**d**) rosmarinic acid. Every C atom in the molecules is numbered to clarify the identification of the signals.

The structure and regioselectivity of the main xylobiosides obtained in the reactions with vanillyl alcohol, phloretin, and rosmarinic acid were determined by NMR. These glycosides were selected based on their high yields, ease to purify, and biotechnological interest. Their deduced glycoside structures are depicted in Figure 6 and the corresponding chemical shifts in Tables S2–S4. In the case of vanillyl alcohol xylobioside, the glycosynthase attached the xylobiosyl residue to the aromatic hydroxyl group of vanillyl alcohol (Figure 6a), as deduced from the low field shift of the xylobiose reducing end anomeric proton H1 in β configuration (Figure S10) and the long-range coupling observed in the HMBC spectrum between this H1 and the aromatic carbon C4 (Figure S10b), rendering a 4-vanillyl alcohol xylobioside (4-*O*-β-d-xylobiosyl-vanillyl alcohol). For the phloretin xylobioside, the main isolated product presented the xylobiose moiety attached to the phenol ring (Figure 6b), as deduced from the observation of correlations in the ROESY spectrum from the xylobiose anomeric H1 to the protons in positions 3 and 5 in phloretin (Figure S11), yielding a 4-phloretin xylobioside (4-*O*-β-d-xylobiosyl-phloretin). Finally, the two major fractions isolated from the reaction with rosmarinic acid were deduced to have the xylobiose molecule attached to the caffeic moiety of rosmarinic acid, at position 4 in xylobioside (1) (Figure 5c), and at position 3 for xylobioside (2) (Figure 5d). In rosmarinic acid xylobioside (1), a ROE crosspeak between the anomeric H1 of xylobiose and the proton on carbon 5 of rosmarinic acid (Figure S12) confirms the regiochemistry of 4-rosmarinic acid xylobioside (4-*O*-β-d-xylobiosyl-rosmarinic acid). For the rosmarinic acid xylobioside (2), the ROE correlation occurred between the anomeric H1 of xylobiose and the proton on carbon 2 of rosmarinic acid (Figure S13), supporting the production of 3-rosmarinic acid xylobioside (3-*O*-β-d-xylobiosyl-rosmarinic acid).

The glycosylation of phenolic antioxidants is of biotechnological interest, as it has been shown to increase their solubility, which may lead to a higher bioavailability and better bioactive properties [54]. In this sense, previous studies have described the glucosylation of some of the phenolic compounds tested in this work and its positive effect on their properties. For instance, the glucoside of pterostilbene was proven to improve the solubility of the original aglycone and to reduce its toxicity for several human cell lines [49], while the glucosylation of phloretin also led to a higher solubility and a lower skin penetrability, which could favor a prolonged protection of the external skin layers by cosmetic preparations [55]. Nevertheless, very few cellobiosides of phenolic compounds obtained through enzymatic synthesis can be found. Still, this type of glycosylation was demonstrated to significantly increase the solubility and stability of hydroquinone, methyl gallate, ethyl gallate, propyl gallate, and epicatechin [56]. The enzymatic synthesis and applications of xylosides of phenolic compounds have also received less attention compared with the glucoside ones. Among the published data, the xyloside of hydroxytyrosol produced by the β-xylosidase BxTW1 from *T. amestolkiae* showed an enhancement of its neuroprotective capacity and antioxidant activity [57]. The thioglycoligase derived from the enzyme used in this last study was also able to xylosylate a broad range of phenolic compounds including gallic acid, EGCG, phloretin, and pterostilbene [58]. Regarding xylobiosides of phenolic compounds, very little is known about their enzymatic synthesis and improved biological properties other than being a dihydroresveratrol xylobioside produced by chemical synthesis and acting as an effective melanogenesis activator [59].

## 3. Materials and Methods

### 3.1. Strains and Media

The fungus *T. amestolkiae*, isolated from cereal wastes and deposited in the IJFM culture collection at “Centro de Investigaciones Biológicas Margarita Salas” (Madrid, Spain) with reference A795, was maintained in potato dextrose agar (PDA, Becton Dickinson, Franklin Lakes, NJ, USA) plates. *Escherichia coli* DH5α (Invitrogen, Waltham, MA, USA) was used for cloning and plasmid propagation and was cultivated in LB medium [60]. *P. pastoris* GS115 (Invitrogen) was employed for the heterologous expression of the *T. amestolkiae* β-1,4-endoxylanase (rXynSOS) and its glycosynthase variants. The wild-type strain was grown in YPD medium [60].

### 3.2. Nucleic Acid Isolation and Cloning of rXynSOS

The nucleotide sequence of *xynSOS* (GenBank accession No. RAO73501.1) was retrieved from the genome of *T. amestolkiae*, which was previously sequenced and annotated [25]. The presence of the signal peptide in the XynSOS protein sequence was examined using the SignalP 5.0 server [61].

For RNA isolation, *T. amestolkiae* was grown for 8 days at 28 °C and 250 rpm (Innova 44 incubator shaker, New Brunswick Scientific, Enfield, CT, USA) in Mandels medium [25] with 1% (*w*/*v*) microcrystalline cellulose (Merck, Kenilworth, NJ, USA) as a carbon source and filtered through 0.8 μm membrane filters (Millipore, Burlington, MA, USA). Then, the RNA was extracted using the RNeasy Plant Mini Kit (Qiagen, Hilden, Germany), and an RT-PCR was carried out to synthesize cDNA with Superscript II Reverse Transcriptase (Invitrogen) according to the manufacturer’s instructions. PCR amplification of *xynSOS* nucleotide sequence, excluding the signal peptide region, was performed using primers including *EcoR*I and *Not*I restriction sites (underlined) (rXynSOS fw 5′-GAATTCCAATTGAATACCGCCGCAAA-3′, rXynSOS rv 5′-GCGGCCGCTTACAAACATTGAGAGTAGTATGG-3′). The PCR product obtained was digested with the corresponding restriction enzymes (New England Biolabs) and introduced in the pPIC9 expression vector (Invitrogen) by ligation using T4 DNA ligase (Promega, Madison, WI, USA). Plasmids were propagated in *E. coli* DH5α via heat shock transformation protocol. Positive clones were selected in LB medium plates containing 100 mg/L ampicillin and plasmids were isolated with the High Pure Plasmid Isolation Kit (Roche, Basel, Switzerland). After confirming the correct sequence, the vector was linearized with *Sal*I (New England Biolabs, Ipswich, MA, USA) and used to transform *P. pastoris* GS115. The electroporation protocol described by the manufacturer was followed utilizing pulses of 1.5 kV, 25 μF, and 200 Ω. Transformants were selected in histidine deficient YNB glucose medium plates prepared according to the manufacturer’s protocol (Becton Dickinson) and later cultured in 20 mL YEPS medium [60] for 7 days at 28 °C and 250 rpm with daily addition of 6.5 mL/L methanol to identify the highest rXynSOS-producing mutants. Samples were taken daily to determine rXynSOS production by measuring OD_600_ and endoxylanase activity against azurine cross-linked (AZCL)–beechwood xylan (Megazyme, Wicklow, Ireland).

### 3.3. Conversion of rXynSOS into Its Glycosynthase Variants

The vector pPIC9 containing the *xynSOS* gene was used as a template to develop two variants of the enzyme by directed mutagenesis. The glutamic acid at position 236 (E236), located in the catalytic site, was replaced by glycine or serine. The identification of the catalytic amino acids of rXynSOS was performed by alignment using Clustal Omega [62] with the GH10 endoxylanases sequences of the fungi *Penicillium simplicissimum* [63], *Aspergillus nidulans* [64], and *Thermoascus aurantiacus* [65], in which the catalytic role for these amino acids had been previously postulated by crystallography. For the mutagenic PCR, the Expand™ Long Template PCR System (Roche) was used as described by the manufacturer. The primers rXynSOS-Gly fw (5′-TAATGTTGCCATCACTGGTCTCGACATCCGTATGA-3′), rXynSOS-Gly rv (5′-TCATACGGATGTCGAGACCAGTGATGGCAACATTA-3′), rXynSOS-Ser fw (5′-TAATGTTGCCATCACTTCTCTCGACATCCGTATGA-3′), and rXynSOS-Ser rv (5′-TCATACGGATGTCGAGAGAAGTGATGGCAACATTA-3′) were employed for the glycine and serine replacements, respectively. The PCR product generated was finally digested by *Dpn*I (New England Biolabs) in order to hydrolyze the parental methylated DNA used as template. Following the same procedure as specified in Section 3.2, *P. pastoris* GS115 was transformed with the new vectors, and the highest rXynSOS glycosynthases producers were selected. In this case, enzyme production was determined by measuring the total extracellular protein concentration with Bradford assays (Bradford Protein Assay Kit, Bio-Rad, Hercules, CA, USA) using bovine serum albumin (BSA, Sigma-Aldrich, St. Louis, MO, USA) as standard, since hydrolytic activity was no longer detected in these mutants.

### 3.4. Production and Purification of rXynSOS and Its Glycosynthase Variants

The selected *P. pastoris* clones were grown in triplicate in 1 L flasks with 200 mL of YEPS medium to produce recombinant enzymes. Cultures were incubated for 7 to 9 days at 28 °C and 250 rpm with daily addition of 6.5 mL/L methanol. Growth was monitored by measuring OD_600_. Endoxylanase rXynSOS activity was assayed against 1% (*w*/*v*) AZCL-beechwood xylan. The reactions (200 µL) were incubated at 50 °C and 1200 rpm for 10 min (TS-100 thermo-shaker, Biosan, Riga, Latvia), stopped by adding 500 μL of 4% (*w*/*v*) Tris buffer pH 10, and centrifuged for 5 min at 20,000× *g* and room temperature (RT) (Centrifuge 5424, Eppendorf, Hamburg, Germany) to remove the insoluble AZCL-xylan. The absorbance of the supernatants containing soluble AZCL-xylooligosaccharides was measured in a spectrophotometer at 590 nm. In the case of the rXynSOS glycosynthase variants, protein production was analyzed by Bradford assays.

For enzyme purification, cultures were harvested and centrifuged for 20 min at 10,000× *g* and 4 °C (Sorvall LYNX 6000 centrifuge, Thermo Scientific, Waltham, MA, USA), and the supernatant was sequentially vacuum filtered through 0.8, 0.45, and 0.22 μm nitrocellulose membrane discs (Millipore). Then, it was concentrated and dialyzed against 10 mM phosphate buffer pH 6 using an ultrafiltration cell (Amicon, Millipore) with a 10 kDa cutoff polysulfone membrane (Millipore). rXynSOS and its glycosynthase variants were purified in a single chromatographic step using an FPLC system (Äkta). The dialyzed crudes were loaded in a 5 mL QFF HiTrap anion exchanger cartridge (Cytiva, Marlborough, MA, USA), equilibrated with 10 mM phosphate buffer pH 6.0, and eluted with a flow of 2 mL/min. rXynSOS and its glycosynthase variants were not retained in the column, eluting before the application of 1 M NaCl in 10 mM phosphate buffer pH 6.0. The purified enzymes were concentrated by ultrafiltration, using 10 kDa cutoff Amicon Ultra-15 centrifugal devices (Millipore), and their concentration was determined by measuring 280 nm absorbance in a Nanodrop spectrophotometer (Thermo Fisher Scientific) and by Bradford assays.

### 3.5. Determination of the Physicochemical Properties of rXynSOS

The estimated molecular mass (*M*_w_) and purity of the enzymes was determined by SDS-PAGE in 10% acrylamide gels [66] stained with Coomassie brilliant blue R-250 (Bio-Rad). Then, the accurate *M*_w_ of rXynSOS was defined by MALDI-TOF using an Autoflex III instrument (Bruker Daltonics, Billerica, MA, USA). The theoretical *M*_w_ from the rXynSOS amino acid sequence was calculated with the ProtParam tool of ExPASy server [67], and the prediction of *N*- and *O*-glycosylation sites was conducted with NetNGlyc 1.0 [68] and NetOGlyc 4.0 [69] servers, respectively. A size exclusion chromatography column (Superdex 75 10/300, Cytiva), previously calibrated with a standard protein kit and equilibrated with 10 mM phosphate buffer pH 6 containing 100 mM NaCl, was used to determine whether rXynSOS was a monomeric or a multimeric enzyme.

The influence of pH and temperature on rXynSOS activity was determined in triplicate reactions (200 μL) using 1% (*w*/*v*) AZCL–beechwood xylan as substrate and 0.1% BSA to prevent activity loss when working with low enzyme concentrations [70]. Optimal pH was evaluated on a range of 2.2 to 7 by using glycine-HCl (pH 2.2–3), sodium formate (pH 3–4), sodium acetate (pH 4–5.5), and histidine-HCl (pH 5.5–7) buffers at a concentration of 50 mM and conducting the reactions at 50 °C. Optimal temperature was assayed in reactions with the substrate in 50 mM sodium acetate buffer pH 5 at temperatures varying from 30 to 80 °C.

The functionality of rXynSOS CBM1 domain toward cellulose was evaluated by incubating the enzyme with 1% (*w*/*v*) microcrystalline cellulose and 0.1% BSA at 4 °C and 1200 rpm. Samples were taken after 10 min, 1 h, and 2 h and centrifuged for 5 min at 20,000× *g* and RT. The endoxylanase activity against AZCL–beechwood xylan was assayed in the supernatants to determine the enzyme concentration bound to cellulose. A control reaction without microcrystalline cellulose was also included, and its activity was considered as 100%. The CBM domain and its assignment to family 1 was predicted by the automated annotator dbCAN2 [71].

### 3.6. Substrate Specificity and Kinetic Assays of rXynSOS

rXynSOS activity was assayed in triplicate against different substrates together with 0.1% BSA. For the *p*-nitrophenol (*p*NP)-based substrates, reactions were conducted using 0.1% (*w*/*v*) 4-nitrophenyl-β-xylobioside (*p*NPX_2_, Megazyme), 4-nitrophenyl β-d-xylopyranoside (*p*NPX, Sigma-Aldrich), 4-nitrophenyl β-d-cellobioside (*p*NPG_2_, Sigma-Aldrich), and 4-nitrophenyl β-d-glucopyranoside (*p*NPG, Sigma-Aldrich) in 50 mM sodium acetate buffer pH 5 (200 μL). After 10 min incubation at 50 °C and 1200 rpm, reactions were stopped by adding 500 μL of 2% (*w*/*v*) Na_2_CO_3_, and the absorbance of the *p*NP released was measured at 410 nm (ε_410_ = 15,200 M^−1^·cm^−1^). One unit of enzymatic activity was defined as the amount of enzyme capable of releasing 1 μmol of *p*NP per min. For the remaining substrates, the reactions were set with 0.8% (*w*/*v*) beechwood xylan (Megazyme, Bray, Ireland), wheat arabinoxylan (Megazyme), carboxymethylcellulose (CMC, Sigma-Aldrich), and microcrystalline cellulose in 50 mM sodium acetate buffer pH 5 (500 μL). The reactions were incubated at 50 °C and 1200 rpm for 10 min and stopped by heating at 100 °C for 5 min. After centrifuging for 5 min at 20,000× *g* and RT, the release of reducing sugars was measured using the Somogyi–Nelson method [72].

The kinetic constants of rXynSOS were studied over different ranges of beechwood xylan (0.06–8 g/L), wheat arabinoxylan (0.12–12 g/L), CMC (4–64 g/L), and *p*NPX_2_ (0.025–2.5 mM), following the reactions settings explained before for each substrate. The *K_m_* and *V*_max_ parameters were calculated using SigmaPlot (Stat-Ease, Minneapolis, MN, USA).

### 3.7. Transglycosylation Assays

The transglycosylation capability of rXynSOS and its glycosynthase variants was evaluated by setting up reactions with different donors and acceptors. The list of acceptors studied included *p*NPX_2_, vanillyl alcohol, 2-hydroxybenzyl alcohol, hydroquinone, gallic acid, and rosmarinic acid, which were purchased from Sigma-Aldrich, and epigallocatechin gallate (EGCG) [73], phloretin [55], and pterostilbene [49], which were provided by Prof. Francisco J. Plou (ICP-CSIC). For the native rXynSOS, 0.1% (*w*/*v*) *p*NPX_2_ was used as xylobiose donor together with 4.6 mg/L of the enzyme and 20 mM of the acceptors. Moreover, 0.1% (*w*/*v*) *p*NPX and *p*NPG_2_ were tested as xylose and cellobiose donors, respectively, using 0.46 g/L catalyst, and when 0.1% (*w*/*v*) *p*NPG was used as the glucose donor, 4.6 g/L enzyme were added to the reactions. All these reactions (100 μL) were adjusted to pH 5 with 50 mM sodium acetate buffer, incubated at 50 °C and 1200 rpm for 1 h, and stopped by heating for 5 min at 95 °C. For the glycosynthase variants rXynSOS-E236G and E236S, 20 mM xylobiose-fluoride (X_2_F) was used as a xylobiose donor together with 4 mg/mL of the enzymes, 20 mM of the acceptors, and 50 mM sodium acetate buffer pH 5. The production of XOS was evaluated with 20 mM X_2_F as substrate and the same rXynSOS-E236G concentration. The use of 20 mM xylose-fluoride (XF), glucose-fluoride (GF), and cellobiose-fluoride (G_2_F) as xylose, glucose, and cellobiose donors, respectively, was also assayed. All fluorinated sugar donors contain the fluorine atom in the α position in carbon 1 of the carbohydrate and were synthesized as previously reported [74]. The reactions (100 μL) were conducted for 16 h at 25 °C and 500 rpm.

To assess the synthesis of glycosides and oligosaccharides, the reactions were analyzed by thin-layer chromatography (TLC) using silica gel G/UV254 polyester plates (Macherey-Nagel). The mobile phases were composed of ethyl acetate:methanol:water in 10:2:1 (*v*/*v*) proportions in the case of glycosides and of butanol:acetic acid:water in 3:1:1 (*v*/*v*) proportions for a better separation of oligosaccharides. Substrates and products were visualized first under 254 nm UV light and then by immersing the plates on a solution of 5% sulfuric acid in methanol and heating 12 min at 100 °C. 

To identify the products spotted by TLC, electrospray ionization-mass spectrometry (ESI-MS) analyses of the corresponding reactions were carried out in a HCT Ultra ion trap (Bruker Daltonics), using methanol as an ionizing phase in the positive reflector mode. The data obtained were processed with the Masshunter Data Acquisition B.05.01 and Masshunter Qualitative Analysis B.07.00 software (Agilent Technologies, Santa Clara, CA, USA). The expected products were detected as sodium adducts of the molecules in most of the cases.

The glycosylation yields of the rXynSOS-E236G reactions obtained with the different acceptors were calculated by HPLC in an Agilent 1200 series LC instrument equipped with a reverse phase ZORBAX Eclipse plus C18 column (Agilent). The column was equilibrated in a mix of acetonitrile (ACN) and H_2_O with 0.1% acetic acid, with a flow of 2 mL/min. The reaction products were separated isocratically, in 8 min, using different percentages of ACN:H_2_O (*v*/*v*), depending on the acceptor: 9:91 for vanillyl alcohol; 10:90 for 2-hydroxybenzyl alcohol, 6:94 for hydroquinone and gallic acid, 13:87 for EGCG, 20:80 for rosmarinic acid, 33:77 for phloretin, and 50:50 for pterostilbene. After elution, the column was washed for 5 min with 95:5 (*v*/*v*) ACN:H_2_O, and the system was finally re-equilibrated to the initial conditions for 3 min. Products were detected monitoring the absorbance at 270 nm and quantified by comparing the peaks areas with the calibration curves of the non-glycosylated acceptors.

### 3.8. Purification and NMR Analysis of Glycosylated Products

The most interesting glycosides synthesized by the rXynSOS-E236G glycosynthase variant were further studied by NMR. Thus, the main glycosides of vanillyl alcohol, phloretin, and rosmarinic acid were purified by HPLC using the settings described in Section 3.7 and later lyophilized. Samples of the purified glycosides, acceptors, X_2_F, and xylobiose were prepared by dissolving the compounds in deuterated water (D_2_O) to concentrations of 1.5–3 mM. NMR spectra were acquired at 298 K, using a Bruker AVIII 600 MHz spectrometer (Bruker Daltonics). Then, 1D ^1^H, 2D ^1^H-^13^C HSQC and HMBC, and ^1^H-^1^H ROESY (300 ms mixing time) and TOCSY (20 and 70 ms mixing time) experiments were performed to assign all NMR signals. For 1D ^1^H, 2D ^1^H-^13^C HSQC, HMBC, DOSY, ROESY, and TOCSY experiments, the standard zg, zgpr, hsqcedetgp, hmbcgpndqf, ledbpgp2s, roesyphpr, and dipsi2phpr sequences included in TOPSPIN software (Bruker Daltonics) were employed. Chemical shifts were referenced to the residual water signal set at 4.77 ppm at 298 K [75].

In order to confirm the regioselectivity of the rXynSOS-E236G glycosynthase variant in the synthesis of XOS, the reaction was directly monitored in an NMR tube at 25 °C by the sequential acquisition of 1D-^1^H spectra. Briefly, a reaction mixture containing 4 mg/mL XynSOS-E236G in 50 mM sodium acetate buffer pH 5 was prepared in the NMR tube (3 mm diameter). The tube was introduced in the spectrometer, and 25 mM X_2_F were added up to 200 μL. Then, the reaction was followed by acquiring 1D-^1^H spectra at different time points (5 min, 1 h, 3 h, and 8 h after X_2_F addition). The spectra of the XOS synthesis reaction mixture were compared with the spectrum of commercial 1,4-β-d-xylotetraose (Megazyme).

## 4. Conclusions

A novel GH10 β-1,4-endoxylanase (XynSOS) from the ascomycetous fungus *T. amestolkiae* has been successfully produced in *P. pastoris*. This versatile enzyme with high affinity for beechwood xylan and wheat arabinoxylan was able to transglycosylate phenolic compounds although with low efficiencies. The replacement of rXynSOS nucleophile residue E236 by a glycine resulted in a glycosynthase variant (rXynSOS-E236G) that represents a promising catalyst to synthesize XOS of four, six, eight, and ten xylose units and to efficiently glycosylate a broad range of phenolic compounds with biotechnological applications. To the best of our knowledge, this is the first time that xylobiosides of phenolic antioxidants are enzymatically synthesized by a fungal glycosynthase. Considering all the benefits that glycosylation can have on the bioavailability and bioactive properties of phenolic compounds, these findings are of great interest to maximize the production of xylobiosides and to study their potential applications. Furthermore, a new horizon is opened to analyze whether xylobiosides have similar or better effects than glucosides, cellobiosides, and xylosides on the biological performance of phenolic antioxidants.

## Figures and Tables

**Table 1:** Endoxylanase-specific activities of rXynSOS against different substrates.

Substrate	Specific Activity (U mg^−1^)
Beechwood xylan (0.8%)	132.33 ± 11.6
Wheat arabinoxylan (0.8%)	149.15 ± 3.45
CMC (0.8%)	1.25 ± 0.35
Microcrystalline cellulose (0.8%)	Not active
Cellobiose (0.1%)	Not active
*p*NPX_2_ (0.1%)	56.97 ± 3.96
*p*NPX (0.1%)	1.81 ± 0.06
*p*NPG_2_ (0.1%)	0.82 ± 0.01
*p*NPG (0.1%)	0.03 ± 0.001

**Table 2:** Kinetic parameters of rXynSOS with different substrates.

Substrate	*K_m_* (g L^−1^)	*k_cat_* (s^−1^)	*k_cat_*/*K_m_* (s^−1^ g^−1^ L)
Beechwood xylan	1.07	144.25	134.69
Wheat arabinoxylan	1.94	163.93	84.65
CMC	51.29	7.90	0.15
*p*NPX_2_	0.05 g L^−1^ 0.12 mM	102.96	2181.45 s^−1^ g^−1^ L 880.04 mM^−1^

**Table 3:** Synthesis of glycosides by the glycosynthase variant rXynSOS-E236G using 20 mM X_2_F as xylobiose donor and different acceptor molecules at 20 mM. Glycosides were detected by ESI-MS in the positive mode as Na-adducts. The yield of the main glycoside of each reaction was calculated by HPLC.

Glycoside	Mass ESI-MS (*m*/*z*) ^1^	Yield HPLC (%)
Vanillyl alcohol-X_2_	441.1	4.3
2-hydroxybenzyl alcohol-X_2_	411.0	7.6
Hydroquinone-X_2_	397.0	26.0
Gallic acid-X_2_	457.1	21.7
Epigallocatechin gallate (EGCG)-X_2_	745.2	1.8
Rosmarinic acid-X_2_ (two main glycosides detected)	647.2	20.9 (1) 20.0 (2)
Phloretin-X_2_	561.1	7.8
Pterostilbene-X_2_	543.3	1.6

^1^ Detected as Na-adducts.

## Data Availability

*T. amestolkiae* whole genome shotgun sequencing project is available at https://www.ncbi.nlm.nih.gov/nuccore/MIKG00000000 (accessed on 24 January 2022).

## Supplementary Materials

The following supporting information can be downloaded at: https://www.mdpi.com/article/10.3390/ijms23031383/s1.
