# Supplementary material for: A Fungal Versatile GH10 Endoxylanase and Its Glycosynthase Variant: Synthesis of Xylooligosaccharides and Glycosides of Bioactive Phenolic Compounds
Source: Int J Mol Sci. 2022 Jan 26;23(3):1383. doi: 10.3390/ijms23031383 (PMC8836076; doi:10.3390/ijms23031383)
Supplement: Supplementary file 1 [file ijms-23-01383-s001.zip › ijms-1552721-supplementary.pdf]

SUPPLEMENTARY INFORMATION

a.

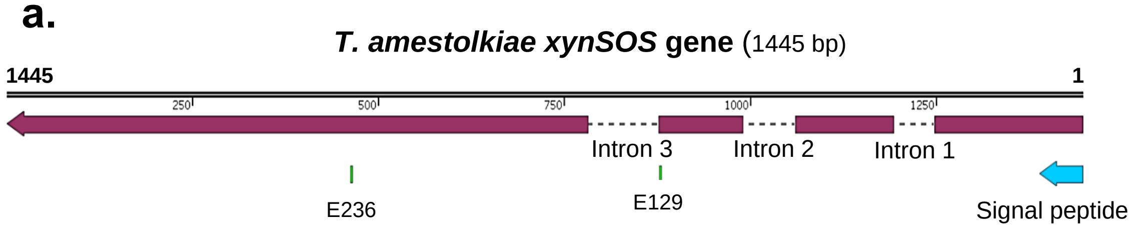

b.

atgactctagtaaagtctattctactagcccttgcggcgggccacgttgcccaggctcaatt
gaataccgccgcaaaagcagctggtttgttgtactttggtactgctgttgacaatccagact
tgagcgactctcaatatattgccaaccttgagaccgcagatttcggtcaaatcacaccagca
aatgcaatgaaagttagtgacggaatttaactaccgtatacattaagctaagcgatgacaat
ttagtggcaacccacggagccgtcccagggttcctatactttcacgcaaggtgaccaaatcg
cgagcctggccaagtcaaataatgactacttgagatgtcacaacctggtctggtacaaccaa
ttgccatcgtacagtaagcacgcatatcacctcttttgtcacctgatatccacgttagtag
actgattccgtgtttttttagtcacttcgggttcatggacaaacgctaccctcatcgctgcc
ctgaaggagcatatcaatggggtagttacgcattacaagggggcagtgttatgcttgggatgt
tgtgaac**gaa**ggtatgtcacgttatttttaggctccctttcccaacccttacaagtctgtca
tcttcattctgtatagtcacgtcgagctaagtggagtatatagccttgaacgaagacggcac
atatcgtcaaaatgttttctaccaatacatcggcgaggcatacatcccgattgcgtttgctg
ccgctgcagcagctgaccctaatgccaagctgtactacaacgattacaacatcgaatacgct
ggggcaaaagcaactggtgctcagggcattgtaaaattgattcaggctgccggcggtcgtat
cgatggtgtgggtcttcagtctcacttcattgtgggatcaacgcctagtcttgccactcaga
aagcaaatatggcttcattcacagctcttggtgttaatgttgccatcact**gag**ctcgacatc
cgtatgactctcccagaaaccaccgctcttgaaactcagcagtccaccgactatcagaccac
cactactgcctgcgtccagactaaaggttgtgtcggtatcactctatgggactatacagaca
agtactcgtgggttcctggtaccttttctggccagggtgatgcctgtccatgggactcaagc
tacaacaagaagcccgcatactatggtattcttgctggcttgcaatctggcactagttcttc
atcctcaacttccagtaccctaataaccaccacaacgcccactactacctcaagtactacgc
caacaactaccacaaccactacctcaggcacaagtgctgcacactggggacaatgcggaggt
atcggctggkctggtcctacgacttgcgtttcaccttacacttgtcaagtgttgaacccata
ctactctcaatgtttgtaa

Figure S1. DNA sequence of *xynSOS* from *T. amestolkiae*. a) Scheme of *xynSOS* gene structure. The coding sequence is represented in purple, the introns in dot lines, the predicted signal peptide in blue and the codons of the acid/base (E129) and nucleophile (E236) catalytic residues in green. b) Sequence of *xynSOS* gene. Introns are indicated in grey, the predicted signal peptide is underlined and the codons of the acid/base (E129) and nucleophile (E236) catalytic residues are highlighted in green.

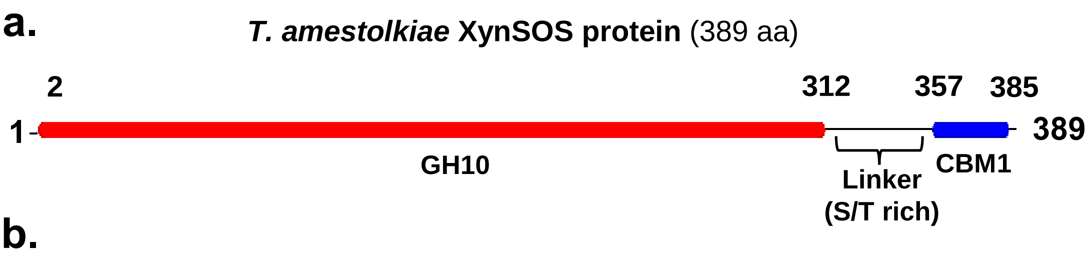

b.

```
MTLVKSILLALAAGHVAQAQLNTAAKAAGLLYFGTAVDNPDLSDSQYIANLETADFGQITPANA
MKWQPTEPSQGSYTFTQGDQIASLAKSNNDYLRCHNLVWYNQLPSYITSGSWTNATLIAALKEH
INGVVTHYKGQCYAWDVVNEALNEDGTYRQNVFYQYIGEAYIPIAFAAAAAADPNAKLYYNDYN
IEYAGAKATGAQGIVKLIQAAGGRIDGVGLQSHFIVGSTPSLATQKANMASFTALGVNVAITEL
DIRMTLPETTALETQQSTDYQTTTTACVQTKGCVGITLWDYTDKYSWVPGTFSGQGDACPWDSS
YNKKPAYYGILAGLQSGTSSSSSTSSTLITTTTPTTTSSTTPTTTTTTTSGTSAAHWGQCGGIG
WSGPTTCVSPYTCQVLNPYYSQCL
```

Figure S2. Protein sequence of XynSOS from *T. amestolkiae*. a) Prediction of CAZy and CBM domains conducted by the dbCAN2 server based on XynSOS amino acid sequence. The predicted GH10 domain is shown in red and the CBM1 domain in blue. In between the GH10 and CBM1 domains a rich Ser/Thr linker region can be found. b) Amino acid sequence of XynSOS. The predicted signal peptide is underlined and the acid/base (E129) and nucleophile (E236) catalytic residues are highlighted in green. Predicted N-glycosylation sites are indicated in pink and O-glycosylation sites in yellow.

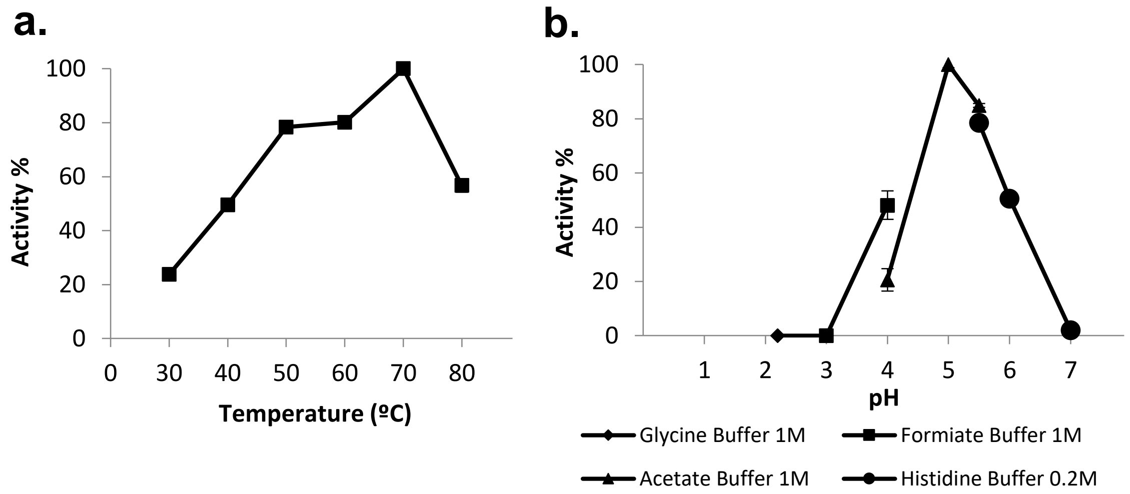

Figure S3. Optimal temperature a) and pH b) of rXynSOS determined with triplicate reactions (200 µL) using 1% (w/v) AZCL-beechwood xylan as substrate and 0.1% BSA to prevent activity loss when working with low enzyme concentrations. The reactions were incubated at 1,200 rpm for 10 min, stopped by adding 500 µL of 4% (w/v) Tris buffer pH 10, and centrifuged to remove the insoluble AZCL-xylan. The absorbance of the supernatants was measured at 590 nm. Optimal pH was evaluated on a range of 2.2 to 7 by using glycine-HCl (pH 2.2-3), sodium formate (pH 3-4), sodium acetate (pH 4-5.5), histidine-HCl (pH 5.5-7) buffers at a concentration of 50 mM and conducting the reactions at 50 ºC. Optimal temperature was assayed in reactions with the substrate in 50 mM sodium acetate buffer pH 5 at temperatures varying from 30 to 80 ºC.

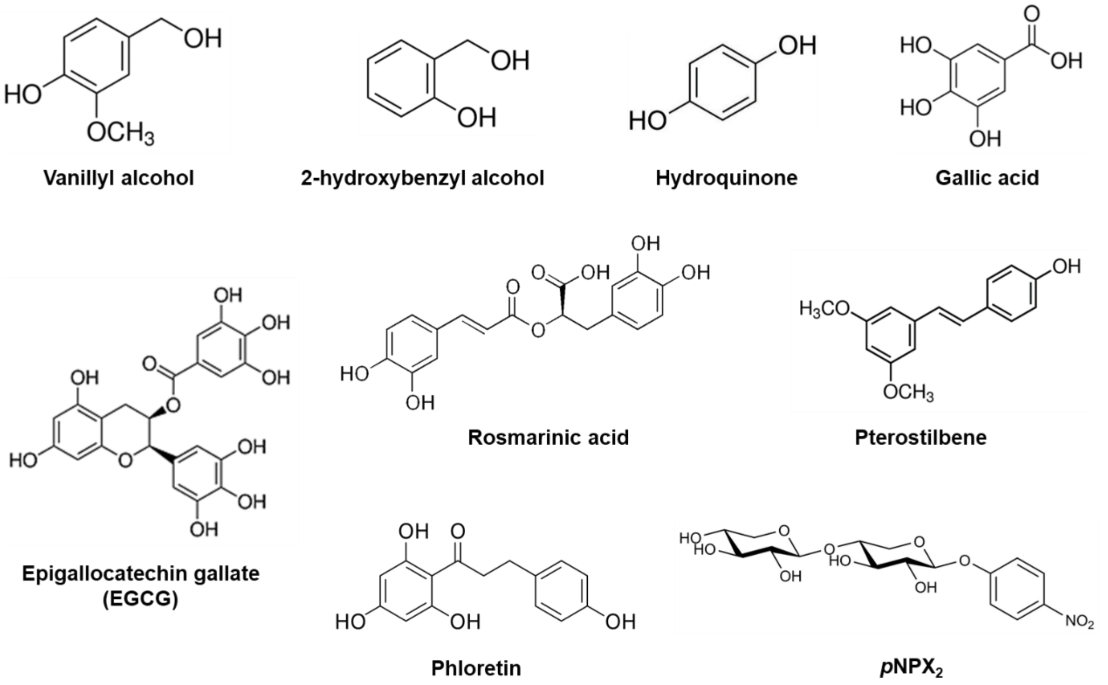

Figure S4. Molecular structure of the acceptor molecules used in the transglycosylation reactions conducted with rXynSOS and/or its glycosynthase variant rXynSOS-E236G.

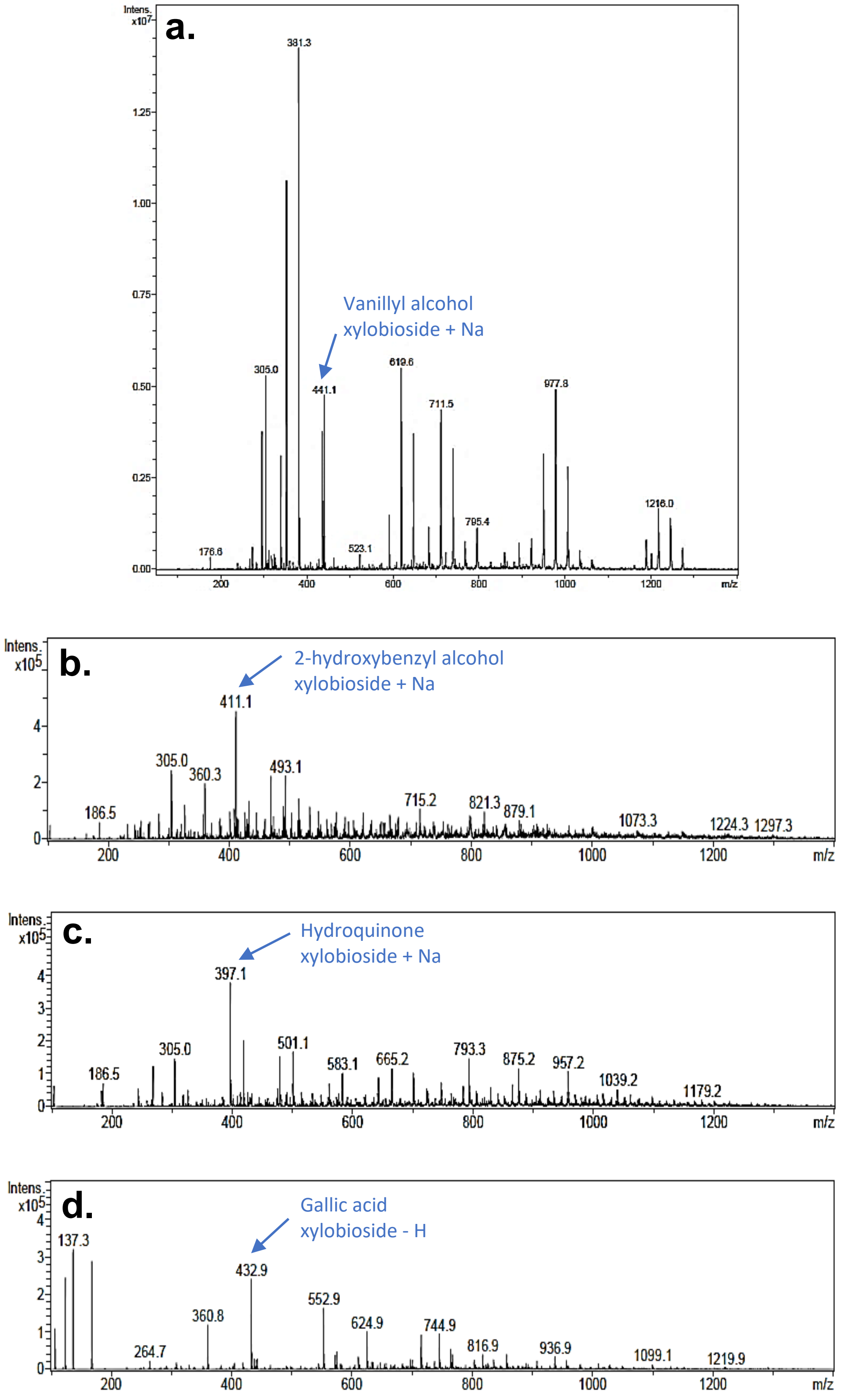

Figure S5. ESI-MS spectra of the transglycosylation reactions conducted by rXynSOS for 1 h at 50 ºC and 1,200 rpm in 50 mM sodium acetate buffer pH 5 with different acceptor molecules at 20 mM and 0.1% (w/v) *p*NPX$_2$ as xylobiose donor (100 µL). Blue arrows show the adducts of the following glycosides: a) vanillyl alcohol; b) 2-hydroxybenzyl alcohol; c) hydroquinone and d) gallic acid.

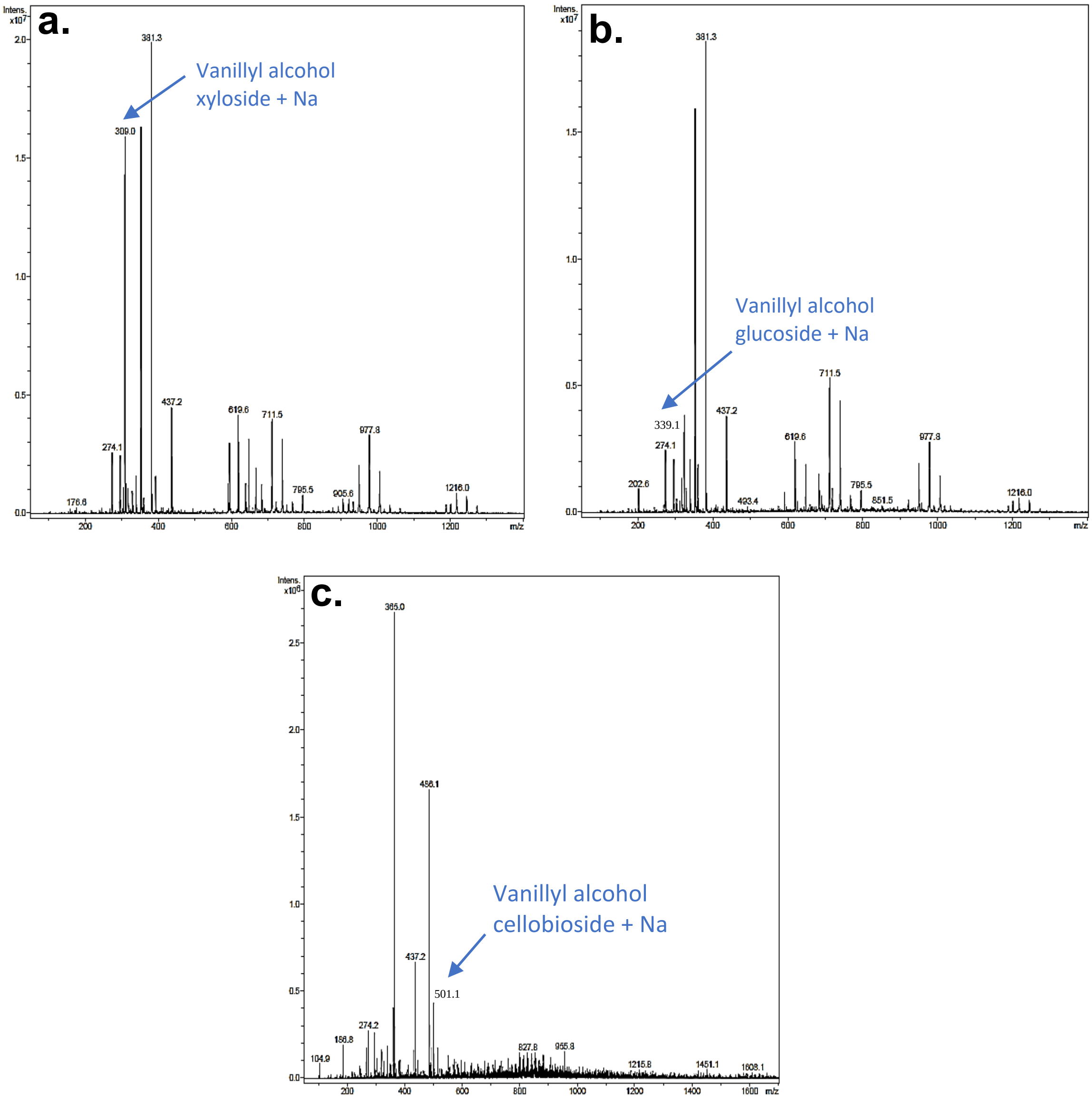

Figure S6. ESI-MS spectra of the transglycosylation reactions conducted by rXynSOS for 1 h at 50 ºC and 1,200 rpm in 50 mM sodium acetate buffer pH 5 with different sugar donors at 0.1% (w/v) and 20 mM vanillyl alcohol as acceptor (100 µL). Blue arrows indicate the Na-adducts of vanillyl alcohol glycosides obtained with a) *p*NPX as xylose donor; b) *p*NPG as glucose donor and c) *p*NPG$_2$ as cellobiose donor.

```
T.amestolkiae|XynSOS              ----QLNTAAKAAGLLYFGTAVDNPDLSDSQYIANLETADFGQITPANAMKWQPTEPSQG 56
A.nidulans|PDBID-1TA3:B           -QSASLNDLFVAAGKSYFGTCSDQALLQ-NSQNEAIVASQFGVITPENSMKWDALEPSQG 58
P.simplicissimum|PDBID-1B30:A     EASVSIDAKFKAHGKKYLGTIGDQYTLTKNTKNPAIIKADFGQLTPENSMKWDATEPNRG 60
T.aurantiacus|PDBID-1K6A:A        QAAQSVDQLIKARGKVYFGVATDQNRLTT-GKNAAIIQADFGQVTPENSMKWDATEPSQG 59
                                      .::    * *  *:*.  *:  *        :  ::** :** *:***:  **.:*

T.amestolkiae|XynSOS              SYTFTQGDQIASLAKSNNDYLRCHNLVWYNQLPSYITSGSWTNATLIAALKEHINGVVTH 116
A.nidulans|PDBID-1TA3:B           NFGWSGADYLVDYATQHNKKVRGHTLVWHSQLPSWVSSIG-DANTLRSVMTNHINEVVGR 117
P.simplicissimum|PDBID-1B30:A     QFTFSGSDYLVNFAQSNGKLIRGHTLVWHSQLPGWVSSIT-DKNTLISVLKNHITTVMTR 119
T.aurantiacus|PDBID-1K6A:A        NFNFAGADYLVNWAQQNGKLIRGHTLVWHSQLPSWVSSIT-DKNTLTNVMKNHITTLMTR 118
                                  .: :: .* :.. * .:.. :* *.***:.***.:::*      **  .:.:**. :: :

T.amestolkiae|XynSOS              YKGQCYAWDVVNEALNEDGTYRQNVFYQYIGEAYIPIAFAAAAAADPNAKLYYNDYNIEY 176
A.nidulans|PDBID-1TA3:B           YKGKIMHWDVVNEIFNEDGTFRNSVFYNLLGEDFVRIAFETARAADPDAKLYINDYNLDS 177
P.simplicissimum|PDBID-1B30:A     YKGKIYAWDVLNEIFNEDGSLRNSVFYNVIGEDYVRIAFETARSVDPNAKLYINDYNLDS 179
T.aurantiacus|PDBID-1K6A:A        YKGKIRAWDVVNEAFNEDGSLRQTVFLNVIGEDYIPIAFQTARAADPNAKLYINDYNLDS 178
                                  ***:   ***:** :****: *:.** : :** :: *** :* :.**:**** ****::

T.amestolkiae|XynSOS              AGAKA-TGAQGIVKLIQAAGGRIDGVGLQSHFIVGSTPSLATQKANMASFTALGVNVAIT 235
A.nidulans|PDBID-1TA3:B           ASYAKTQAMASYVKKWLAEGVPIDGIGSQAHYSSSHWSSTEAAGALSSLANTGVSEVAIT 237
P.simplicissimum|PDBID-1B30:A     AGYSKVNGMVSHVKKWLAAGIPIDGIGSQTHLGAGAG--SAVAGALNALASAGTKEIAIT 237
T.aurantiacus|PDBID-1K6A:A        ASYPKTQAIVNRVKQWRAAGVPIDGIGSQTHLSAGQG--AGVLQALPLLASAGTPEVAIT 236
                                  *.     .  . **   * *  ***:* *:*   .      .  *     .:   ::***

T.amestolkiae|XynSOS              ELDIRMTLPETTALETQQSTDYQTTTTACVQTKGCVGITLWDYTDKYSWVPGTFSGQGDA 295
A.nidulans|PDBID-1TA3:B           ELDIA----------GAASSDYLNLLNACLNEQKCVGITVWGVSDKDSWRASD-----SP 282
P.simplicissimum|PDBID-1B30:A     ELDIA----------GASSTDYVNVVNACLNQAKCVGITVWGVADPDSWRSSS-----SP 282
T.aurantiacus|PDBID-1K6A:A        ELDVA----------GASPTDYVNVVNACLNVQSCVGITVWGVADPDSWRAST-----TP 281
                                  ***:               :** . .**::   *****:*. :*  **  .

T.amestolkiae|XynSOS              CPWDSSYNKKPAYYGILAGLQSGTSSSSSTSSTLITTTTPTTTSSTTPTTTTTTTSGTSA 355
A.nidulans|PDBID-1TA3:B           LLFDGNYQPKDAYNAIVNALS--------------------------------------- 303
P.simplicissimum|PDBID-1B30:A     LLFDGNYNPKAAYNAIANAL---------------------------------------- 302
T.aurantiacus|PDBID-1K6A:A        LLFDGNFNPKPAYNAIVQDLQQ-------------------------------------- 303
                                    :*..:: * ** .*   *

T.amestolkiae|XynSOS              AHWGQCGGIGWSGPTTCVSPYTCQVLNPYYSQCL   389
A.nidulans|PDBID-1TA3:B           ----------------------------------   303
P.simplicissimum|PDBID-1B30:A     ----------------------------------   302
T.aurantiacus|PDBID-1K6A:A        ----------------------------------   303
```

Figure S7. Alignment of XynSOS (*T. amestolkiae*) amino acid sequence excluding its signal peptide with the GH10 endoxylanases sequences of the fungi *Aspergillus nidulans* (PDB ID 1TA3:B), *Penicillium simplicissimum* (PDB ID 1B30:A) and *Thermoascus aurantiacus* (PDB ID 1K6A:A). The catalytic amino acids are highlighted in blue (E129, acid/base residue) and red (E236, nucleophile residue), being the last glutamate the one substituted by glycine and serine. The alignment was performed using Clustal Omega with default settings.

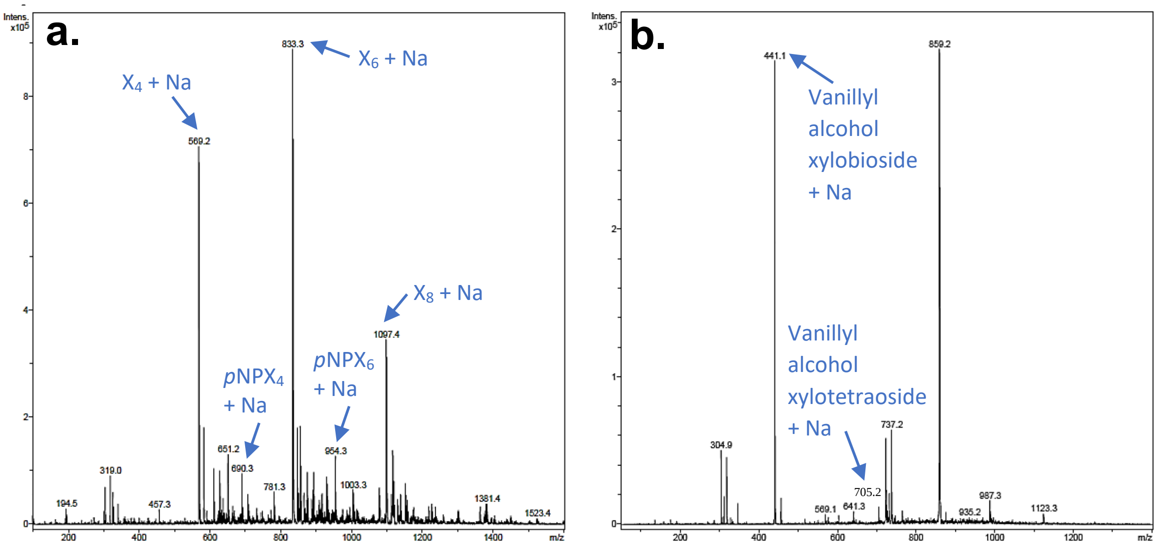

Figure S8. ESI-MS spectra of the glycosylation reactions conducted by rXynSOS-E236G glycosynthase variant for 16 h at 25 ºC and 500 rpm in 50 mM sodium acetate buffer pH 5 using 20 mM X_2F as xylobiose donor and 20 mM *p*NPX$_2$ and vanillyl alcohol as acceptors (100 µL). Blue arrows indicate the Na-adducts of the following glycosides: a) *p*NPX$_2$ and b) vanillyl alcohol. XOS of different lengths (X_4, X_6 and X_8) were detected in the *p*NPX$_2$ reaction a).

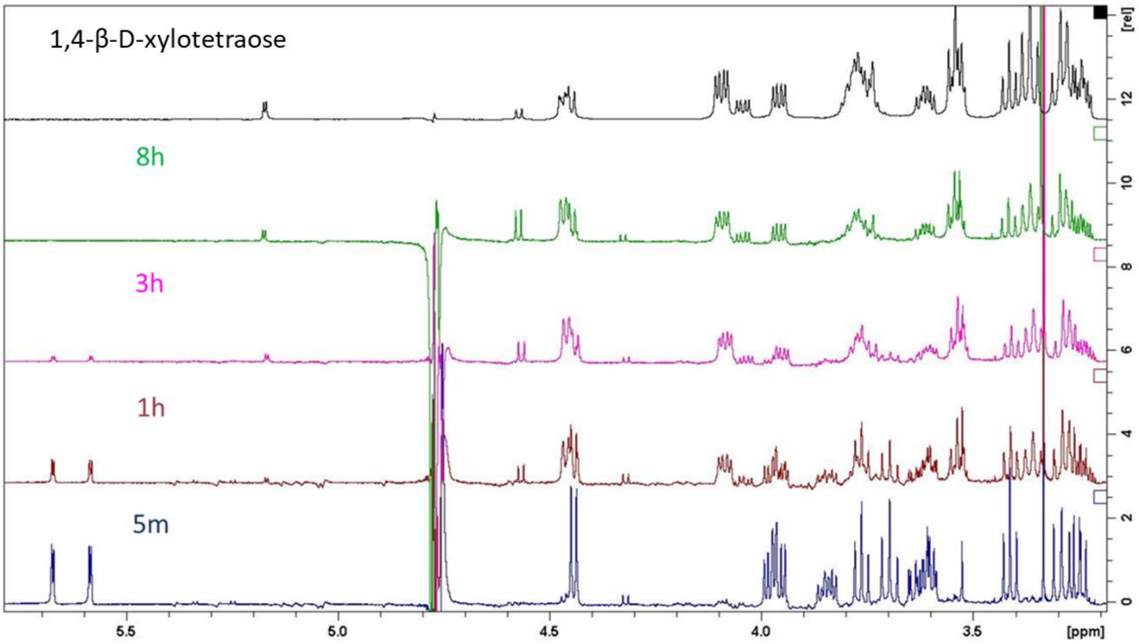

Figure S9. NMR spectra of the XOS synthesis reaction over time. A reaction mixture (200 µL) containing 4 mg/mL XynSOS-E236G in 50 mM sodium acetate buffer pH 5 was prepared in a NMR tube. The tube was introduced in the spectrometer and 25 mM xylobiose-fluoride (X_2F) were added. Series of 1D-^{1}H experiments were then acquired at 298 K at different time points between 5 min and 8 h. The figure displays the spectra of the XOS synthesis reaction acquired after 5 min, 1 h, 3 h and 8 h of X_2F addition and compares them with the spectrum of a sample of commercial 1,4-β-D-xylotetraose. NMR spectra of the monitored reaction in all time points match the one of 1,4-β-D-xylotetraose, confirming the β-1,4 regioselectivity of rXynSOS-E236G glycosynthase variant in XOS synthesis. The dd signal at 5.6 ppm corresponding to H 1 proton coupled with F of X_2F substrate disappeared with time and new signals appeared corresponding to longer xylobioside oligomers with β-1,4 glycosidic bonds.

Table S1. Synthesis of oligosaccharides by rXynSOS-E236G glycosynthase variant in reactions (100 uL) conducted for 16 h at 25 ºC and 500 rpm in 50 mM sodium acetate buffer pH 5 using 20 mM XF, G_2F and GF as xylose, cellobiose and glucose only reactant molecules, respectively. Oligosaccharides were detected by ESI-MS in the positive mode as Na-adducts.

Oligosaccharide	ESI-MS (m/z Na-adduct mass)	Intensity
XF as reactant molecule		
X_2	304.2	245307
X_3	437.2	345614
X_4	569.5	203035
G_2F as reactant molecule		
G_4	689.2	471410
GF as reactant molecule		
Not detected	-	-

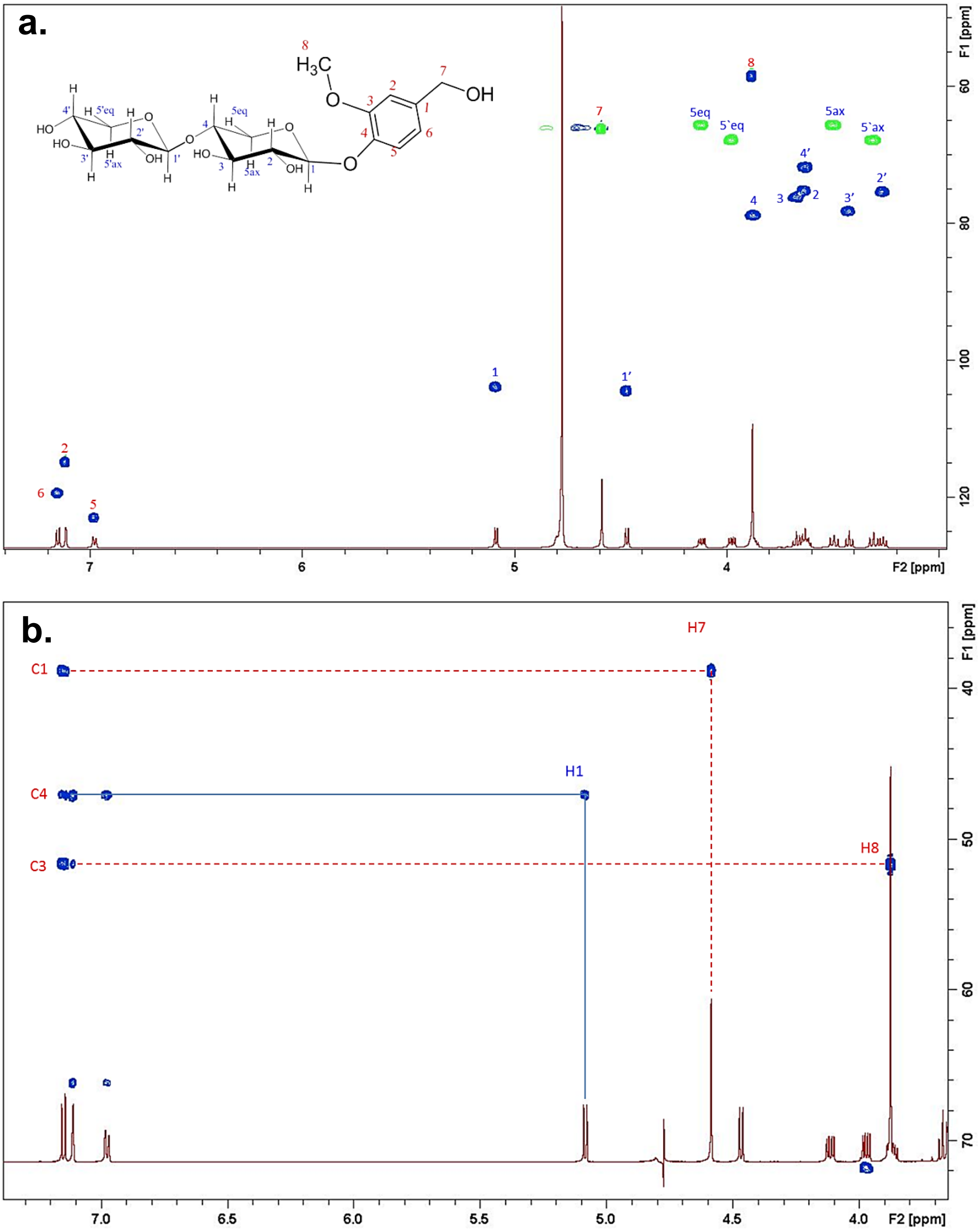

Figure S10. NMR spectra of vanillyl alcohol xylobioside dissolved in deuterated water (D_2O). a) Superimposition of ^{1}H zg (cherry) and ^{1}H-^{13}C HSQC (blue/green) spectra and assignation, xylobiose (blue), vanillyl alcohol (red). b) Superimposition of HMBC (blue) and ^{1}H zgpr (cherry) spectra. The long range ^{1}H-^{13}C correlations showing the connectivity between the xylobiose and vanillyl alcohol are highlighted, disclosing a 4-vanillyl alcohol xylobioside. The HMBC spectrum was acquired with a spectral window of 100 ppm centered at 80 ppm. The peaks of the aromatic carbons appear folded in the upper part of the spectrum shifted 100 ppm.

Table S2. Chemical shifts for vanillyl alcohol xylobioside dissolved in deuterated water (D_2O). Chemical shifts were referenced to the residual water signal set at 4.77 ppm at 298 K.

	^{1}H (ppm)	^{13}C (ppm)
1 X_2	5.08	103.5
2 X_2	3.6	75.2
3 X_2	3.67	76.0
4 X_2	3.8	78.7
5 ax X_2	3.49	65.5
5 eq X_2	4.1	65.68
1’ X_2	4.46	104.49
2’ X_2	3.26	75.3
3’ X_2	3.4	78.18
4’ X_2	3.6	71.7
5’ax X_2	3.3	67.67
5’eq X_2	3.97	67.6

	^{1}H (ppm)	^{13}C (ppm)
**1 Van**		138.7*
**2 Van**	7.11	114.9
**3 Van**		151.6*
**4 Van**		147.2*
**5 Van**	6.9	123.0
**6 Van**	7.15	126.8
**7 Van**	4.58	66.14
**8 Van**	3.87	58.37

*Assignments derived from HMBC spectrum

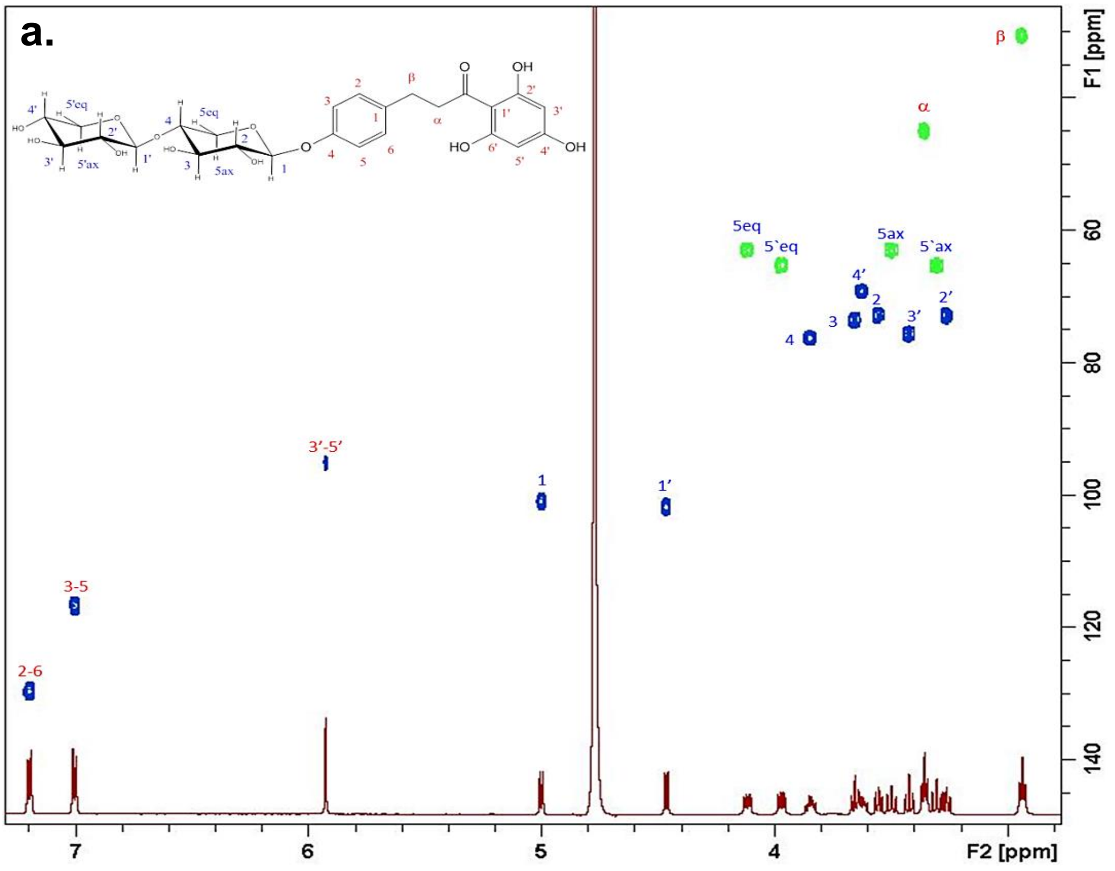

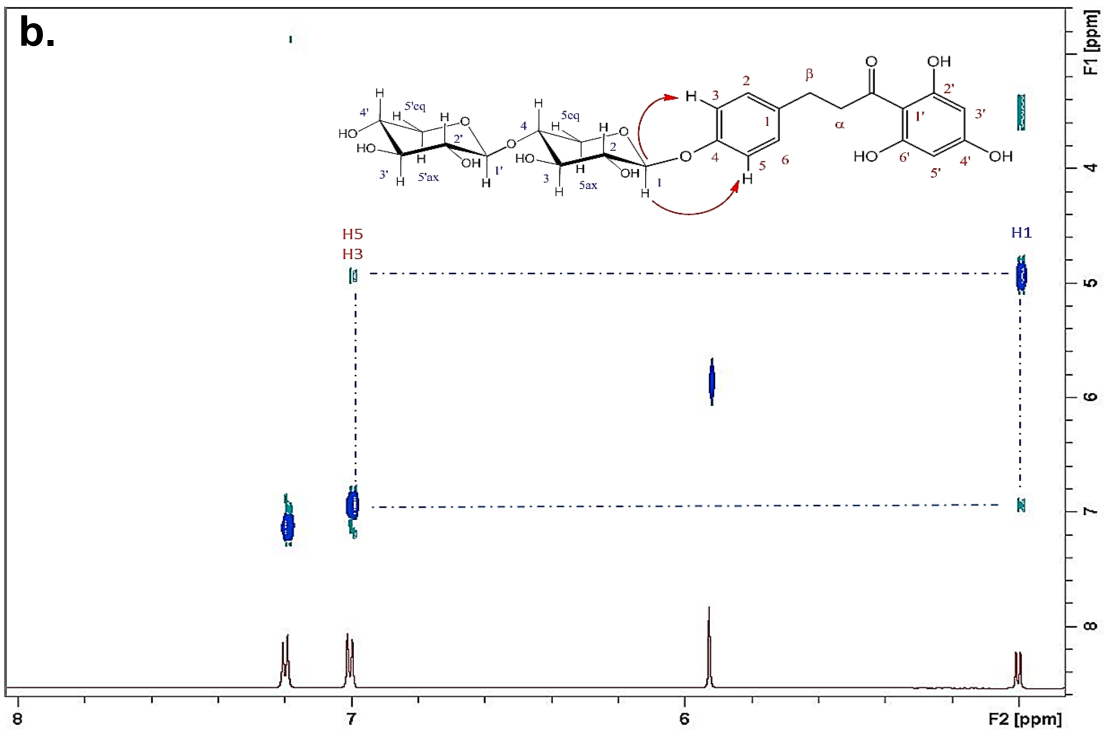

Figure S11. NMR spectra of phloretin xylobioside dissolved in deuterated water (D_2O). a) Superimposition of ^{1}H zg (cherry) and ^{1}H-^{13}C HSQC (blue/green) spectra and assignation, xylobiose (blue), phloretin (red). b) Superimposition of ROESY (blue and green) and ^{1}H zg (cherry) spectra with highlighted ROE correlation between xylobiose ^{1}H 1 and ^{1}H 3 and 5 of phloretin, rendering a 4-phloretin xylobioside.

Table S3. Chemical shifts for phloretin xylobioside dissolved in deuterated water (D_2O). Chemical shifts were referenced to the residual water signal set at 4.77 ppm at 298 K.

	^{1}H (ppm)	^{13}C (ppm)
1 X_2	5.0	100.97
2 X_2	3.55	72.81
3 X_2	3.65	73.58
4 X_2	3.84	76.21
5 ax X_2	3.49	63.11
5 eq X_2	4.12	62.97
1’ X_2	4.46	101.78
2’ X_2	3.26	72.87
3’ X_2	3.42	75.67
4’ X_2	3.62	69.27
5’ax X_2	3.3	65.33
5’eq X_2	3.97	65.32

	^{1}H (ppm)	^{13}C (ppm)
**1 Phlor**		135.4*
**2 (6) Phlor**	7.19	129.66
**3 (5) Phlor**	7.01	116.71
**4 Phlor**		154.6*
**α Phlor**	2.93	30.69
**β Phlor**	3.35	44.9
**C=O Phlor**		206*
**1’ Phlor**		104*
**2’ (6’) Phlor**		163.5*
**3’ (5’) Phlor**	5.93	95.21
**4’ Phlor**		163.5*

*Assignments derived from HMBC spectrum

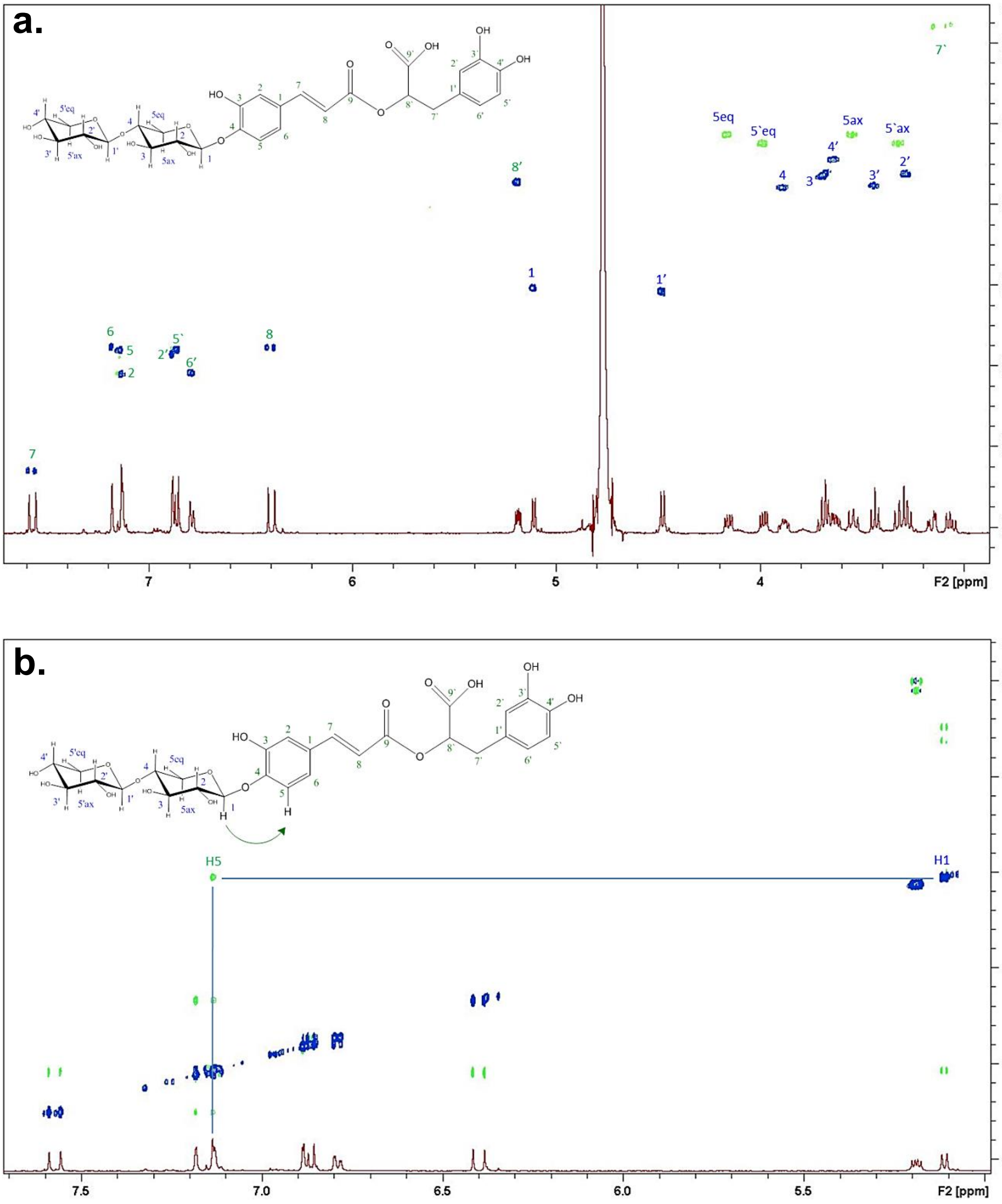

Figure S12. NMR spectra of rosmarinic acid xylobioside (1) dissolved in deuterated water (D_2O). a) Superimposition of ^{1}H zg (cherry) and ^{1}H-^{13}C HSQC (blue/green) spectra and assignation, xylobiose (blue), rosmarinic acid (green). b) Superimposition of ROESY (blue and green) and ^{1}H zg (cherry) spectra with highlighted ROE correlation between xylobiose ^{1}H 1 and ^{1}H 5 of rosmarinic acid (blue line), rendering a 4-rosmarinic acid xylobioside.

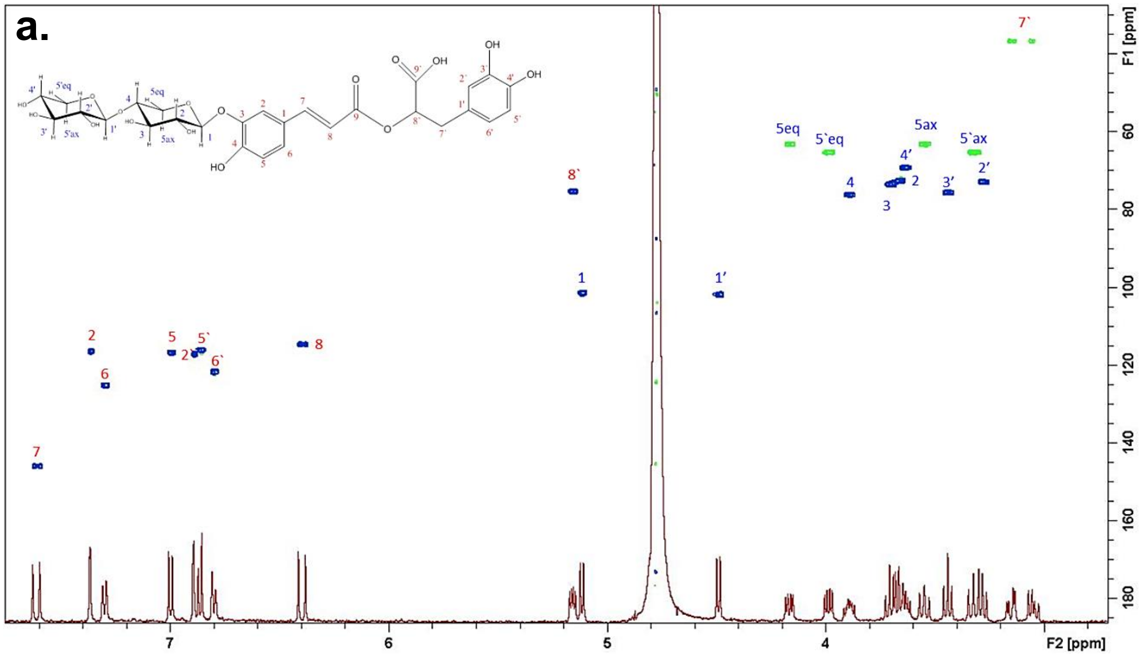

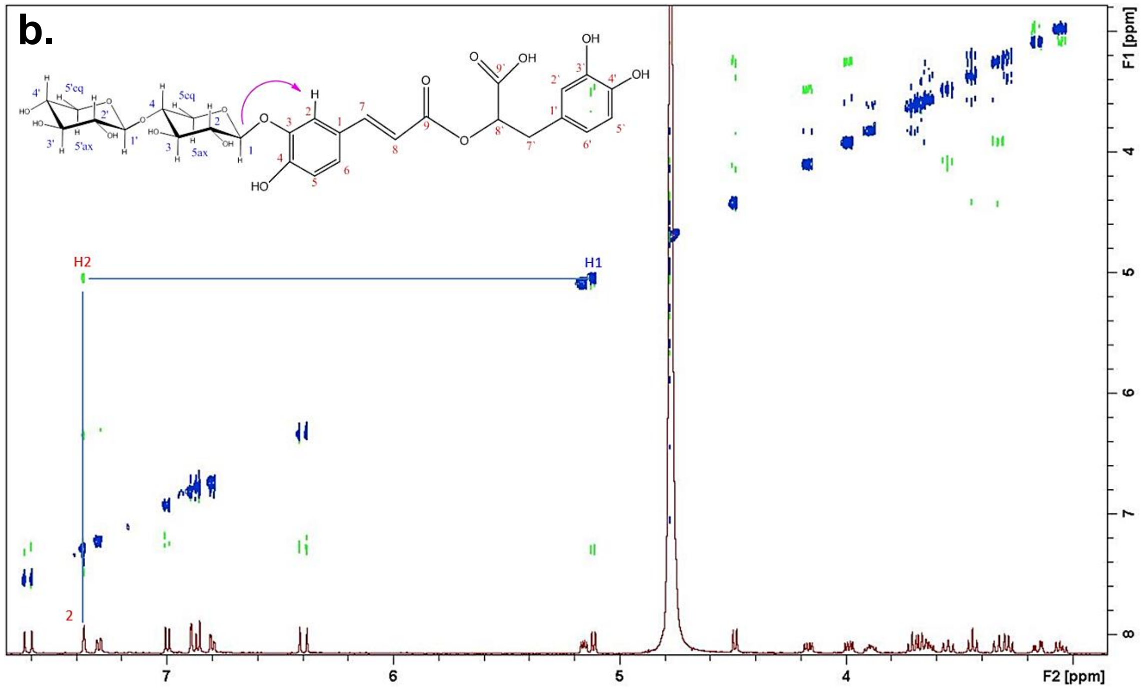

Figure S13. NMR spectra of rosmarinic acid xylobioside (2) dissolved in deuterated water (D_2O). a) Superimposition of ^{1}H zg (cherry) and ^{1}H-^{13}C HSQC (blue/green) spectra and assignation, xylobiose (blue), rosmarinic acid (red). b) Superimposition of ROESY (blue and green) and ^{1}H zg (cherry) spectra with highlighted ROE correlation between ^{1}H 1 and ^{1}H 2 of rosmarinic acid (blue line), rendering a 3-rosmarinic acid xylobioside.

Table S4. Chemical shifts for rosmarinic acid xylobiosides (1) and (2) dissolved in deuterated water (D_2O). Chemical shifts were referenced to the residual water signal set at 4.77 ppm at 298K.

Rosmarinic acid xylobioside (1)

	^1H (ppm)	^{13}C (ppm)
1 X_2	5.12	100.5
2 X_2	3.66	72.2
3 X_2	3.7	73.1
4 X_2	3.9	75.7
5 ax X_2	3.56	64.8
5 eq X_2	4.17	62.6
1' X_2	4.49	101.5
2' X_2	3.2	72.2
3' X_2	3.4	75.1
4' X_2	3.6	68.9
5'ax X_2	3.33	64.6
5'eq X_2	4.0	64.8
**1 Rosm**		129.5*
**2 Rosm**	7.13	122.2
**3 Rosm**		145.5*
**4 Rosm**		146.4*
**5 Rosm**	7.13	116.2
**6 Rosm**	7.18	115.5
**7 Rosm**	7.57	146
**8 Rosm**	6.4	115.4
**9 Rosm**		168.3*
**1' Rosm**		129.1*
**2' Rosm**	6.89	117.3
**3' Rosm**		143*
**4' Rosm**		143.8*
**5' Rosm**	6.79	121.8
**6' Rosm**	6.79	121.8
**7' Rosm**	3.1	35.8
**7' Rosm**	3.2	35.8
**8' Rosm**	5.2	74.4
**9' Rosm**		175.0*

Rosmarinic acid xylobioside (2)

	^1H (ppm)	^{13}C (ppm)
1 X_2	5.11	101.4
2 X_2	3.66	62.6
3 X_2	3.7	73.5
4	3.89	76.0
5 ax X_2	3.5	63.1
5 eq X_2	4.16	63.3
1' X_2	4.5	101.7
2' X_2	3.2	72.8
3' X_2	3.4	75.4
4' X_2	3.6	69.0
5'ax X_2	3.3	65.1
5'eq X_2	3.99	65.1
**1 Rosm**		126.81*
**2 Rosm**	7.36	116.6
**3 Rosm**		144.13*
**4 Rosm**		148.2*
**5 Rosm**	6.99	116.6
**6 Rosm**	7.3	125.12
**7 Rosm**	7.6	145.8
**8 Rosm**	6.39	114.6
**9 Rosm**		168.4*
**1' Rosm**		128.9*
**2' Rosm**	6.89	117.0
**3' Rosm**		143.4*
**4' Rosm**		142.8
**5' Rosm**	6.8	116.0
**6' Rosm**	6.8	121.5
**7' Rosm**	3.14	36.7
**7' Rosm**	3.04	36.7
**8' Rosm**	5.1	75.2
**9' Rosm**		175.8*

*Assignments derived from HMBC spectrum
